# Temporal evolution of the microbiome, immune system and epigenome with disease progression in ALS mice

**DOI:** 10.1242/dmm.041947

**Published:** 2019-11-15

**Authors:** Claudia Figueroa-Romero, Kai Guo, Benjamin J. Murdock, Ximena Paez-Colasante, Christine M. Bassis, Kristen A. Mikhail, Kristen D. Raue, Matthew C. Evans, Ghislaine F. Taubman, Andrew J. McDermott, Phillipe D. O'Brien, Masha G. Savelieff, Junguk Hur, Eva L. Feldman

**Affiliations:** 1 Department of Neurology, University of Michigan, Ann Arbor, MI 48109, USA; 2 Department of Biomedical Sciences, University of North Dakota School of Medicine and Health Sciences, Grand Forks, ND 58202, USA; 3 Department of Internal Medicine, University of Michigan, Ann Arbor, MI 48109, USA; 4 Department of Pharmacology, University of Oxford, Oxford OX1 3QT, UK; 5 Department of Microbiology and Immunology, University of Michigan, Ann Arbor, MI 48109, USA

**Keywords:** Amyotrophic lateral sclerosis, G93A, Gut, Neurodegeneration, SOD1, Immunophenotype

## Abstract

Amyotrophic lateral sclerosis (ALS) is a terminal neurodegenerative disease. Genetic predisposition, epigenetic changes, aging and accumulated life-long environmental exposures are known ALS risk factors. The complex and dynamic interplay between these pathological influences plays a role in disease onset and progression. Recently, the gut microbiome has also been implicated in ALS development. In addition, immune cell populations are differentially expanded and activated in ALS compared to healthy individuals. However, the temporal evolution of both the intestinal flora and the immune system relative to symptom onset in ALS is presently not fully understood. To better elucidate the timeline of the various potential pathological factors, we performed a longitudinal study to simultaneously assess the gut microbiome, immunophenotype and changes in ileum and brain epigenetic marks relative to motor behavior and muscle atrophy in the mutant superoxide dismutase 1 (SOD1^G93A^) familial ALS mouse model. We identified alterations in the gut microbial environment early in the life of SOD1^G93A^ animals followed by motor dysfunction and muscle atrophy, and immune cell expansion and activation, particularly in the spinal cord. Global brain cytosine hydroxymethylation was also altered in SOD1^G93A^ animals at disease end-stage compared to control mice. Correlation analysis confirmed interrelationships with the microbiome and immune system. This study serves as a starting point to more deeply comprehend the influence of gut microorganisms and the immune system on ALS onset and progression. Greater insight may help pinpoint novel biomarkers and therapeutic interventions to improve diagnosis and treatment for ALS patients.

This article has an associated First Person interview with the joint first authors of the paper.

## Abstract (precis)

**Summary:** This study reports associations between symptom onset and the microbiome, immune system and epigenetic marks, establishing a time line that may pinpoint biomarkers of ALS for earlier diagnosis and therapeutic intervention.

## INTRODUCTION

Amyotrophic lateral sclerosis (ALS) is a fatal neurodegenerative disease that affects upper and lower motor neurons, resulting in muscle atrophy, respiratory failure and death (Brown and Al-Chalabi, 2017). ALS risk factors include advanced age and certain genetic mutations; however, epigenetic mechanisms are also altered and may represent a link between genetic factors and life-long environmental exposures, which are also known to increase disease risk (Paez-Colasante et al., 2015; Su et al., 2016). However, despite intense research, the full spectrum and temporal course of ALS risk factors remain unknown, as does the precise disease etiology. Although age, genetics and environmental factors play a role, disease onset likely results from the dynamic interconnectivity of a network of altered pathways over time.

Accumulating evidence suggests that the gut microbiome is important in ALS (McCombe et al., 2019). The intestinal flora in ALS mice expressing mutant human superoxide dismutase 1 (SOD1^G93A^) is distinct from wild-type (WT) animals, with greater intra-communal diversity, differences in specific microbial flora (Blacher et al., 2019) and fewer butyrate-producing bacteria (Wu et al., 2015). Supplementing SOD1^G93A^ animals with bacteria (Blacher et al., 2019) or a bacteria-derived metabolite, e.g. butyrate (Zhang et al., 2017), restores the animal's gut microbiome and lengthens lifespan. Furthermore, clinical studies report differences in fecal microbiota from ALS patients compared to healthy volunteers, suggesting a possible translation to humans (Mazzini et al., 2018; Rowin et al., 2017; Blacher et al., 2019).

Recent studies also implicate the immune system in ALS progression (Thonhoff et al., 2018). As in the microbiome, genetics and epigenome restructuring by environmental cues impact immunity (Anaya et al., 2016). In ALS SOD1^G93A^ mice, immune activation in the central nervous system (CNS) and peripheral nervous system is distinct compared to control animals, with an accumulation of multiple immune cell types associated with disease progression (Alexianu et al., 2001; Chiu et al., 2009). Specific immune cell populations such as CD4T cells may be protective (Beers et al., 2008), whereas others, such as neutrophils, inflammatory monocytes, CD8T cells and innate lymphoid cells, are destructive (Finkelstein et al., 2011; Murdock et al., 2017; Butovsky et al., 2012; Coque et al., 2019). In addition, the impact of the immune system may depend on the stage of disease, with protective cytokines, such as interleukin (IL)-4 and IL-10, expressed in the CNS during early disease, whereas pro-inflammatory cytokines, such as IL-6, tumor necrosis factor alpha (TNF-α) and interferon gamma (IFN-γ), are expressed during late disease (Henkel et al., 2006; Beers et al., 2011). Finally, several studies also report immune changes in the peripheral blood of ALS patients compared to healthy controls. As in mouse models, CD4T cells appear to be protective, whereas other immune cells, such as neutrophils and monocytes, appear to accelerate disease (Murdock et al., 2017; Beers et al., 2018; Gustafson et al., 2017).

There are also emerging relationships among ALS risk factors. For example, the gut microbiome impacts host immunity (Rooks and Garrett, 2016) and vice versa (Kato et al., 2014), suggesting possible mutual regulation. Specifically, the intestines of SOD1^G93A^ mice are populated by an increase in abnormal Paneth cells (Wu et al., 2015). Paneth cells are specialized gut epithelial cells that are part of the host innate immune system and normally release antimicrobial peptides in response to bacterial pathogens. However, in SOD1^G93A^ mice, they are defective and secrete lower levels of antimicrobial peptides, underscoring one mechanism through which the microbiome may influence the immune system in ALS. In addition, as mentioned above, the gut flora of SOD1^G93A^ mice produces less butyrate (Wu et al., 2015), which is a known inducer of differentiation for colonic Treg cells that help maintain tolerance to self-antigens and prevent autoimmune disorders (Furusawa et al., 2013), suggesting another path of convergence between microbiome and immune system.

The microbiome also exerts an effect on the epigenome via secretion of metabolites and immune modulation, even to sites remote from the gut such as the brain (El Aidy et al., 2016), leading to changes in the host epigenome, including DNA methylation, histone modifications and microRNA alterations (Qin and Wade, 2018). In the context of ALS, the gut microbiome-epigenome connection has not been investigated. However, the epigenome is significantly perturbed in ALS, with differences in microRNA, global and loci-specific cytosine methylation (5mC) and hydroxymethylation (5hmC), and histone deacetylase mRNA levels in ALS patient spinal cord tissue compared to healthy individuals (Figueroa-Romero et al., 2016, 2012; Janssen et al., 2010).

Thus, in ALS, the gut microbiome, immune system and epigenome comprise a trio of dysregulated biological processes, the interconnection of which could shed light on disease progression. In the current study, we systematically evaluated alterations in gut microbiome, immune system, and ileum and brain epigenetic (5mC, 5hmC) modifications in SOD1^G93A^ mice at multiple time points relative to symptom onset, i.e. skeletal muscle loss and impaired locomotion. We report new associations between muscle loss and neuromuscular weakness with the microbiome, immunophenotypes and epigenetic marks. Defining the evolution and interrelation of these processes may elucidate ALS pathomechanisms and guide our development of better biomarkers for earlier diagnosis and novel therapies for improved ALS patient survival.

## RESULTS

### SOD1^G93A^ mice exhibit progressive development of ALS symptoms

**Fig. 1.**
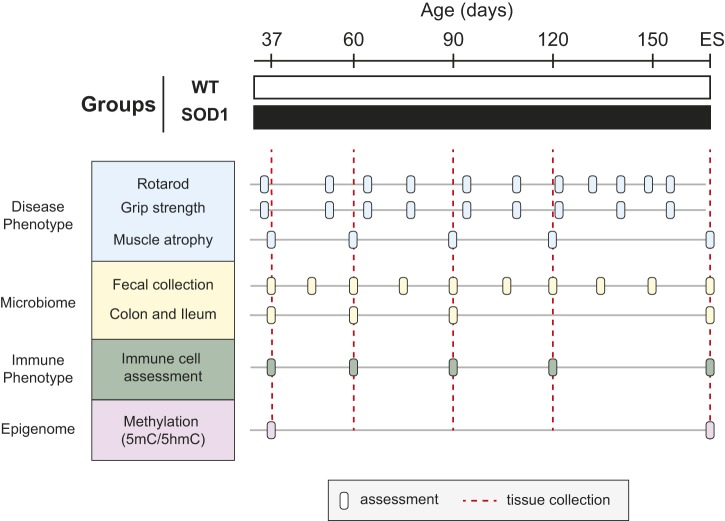
**Study design.** Male and female SOD1^G93A^ and WT control littermates were used to perform a comprehensive longitudinal study for gut microbiome, immune phenotype, and ileum and brain methylation relative to neuromuscular degeneration. Evaluation in SOD1^G93A^ and WT mice. Neuromuscular function (blue): rotarod and grip strength were recorded at 35, 51, 64, 78, 91, 105, 121, 135*, 145, 150* and 155 days (*dates that grip strength was not assessed) and skeletal tissue atrophy was evaluated at 37, 60, 90 and 120 days and end-stage (ES) (*n*=8 mice per group per time point, except the 60-day-old group for which *n*=7). Microbiome evaluation (yellow): colon and ileum content were collected at 37, 60 and 90 days and ES (*n*=8 mice per group per time point, except the 60-day-old group for which *n*=7). Fecal pellets were additionally collected at 45, 75, 105, 120, 135 and 150 days (*n*=all mice alive at that time point). At ES, *n*=8 mice per group. Immune system assessment (green) was performed on bone marrow, blood, spleen, spinal cord and brain at 37, 60, 90 and 120 days and ES (*n*=8 mice per group per time point). Epigenome evaluation (purple) on brain methylation (5mC/5hmC) was evaluated at 37 days and ES and on ileum at ES. The number of animals (*n*) per group (SOD1^G93A^/WT) at each time point for 5hmC was: ileum at ES=7/6; brain at 37 days=7/8 and at ES=7/6. The number of animals (*n*) per group (SOD1^G93A^/WT) at each time point for 5mC was: ileum at ES=7/7; brain at 37 days=7/8 and at ES=7/6.

**Fig. 2.**
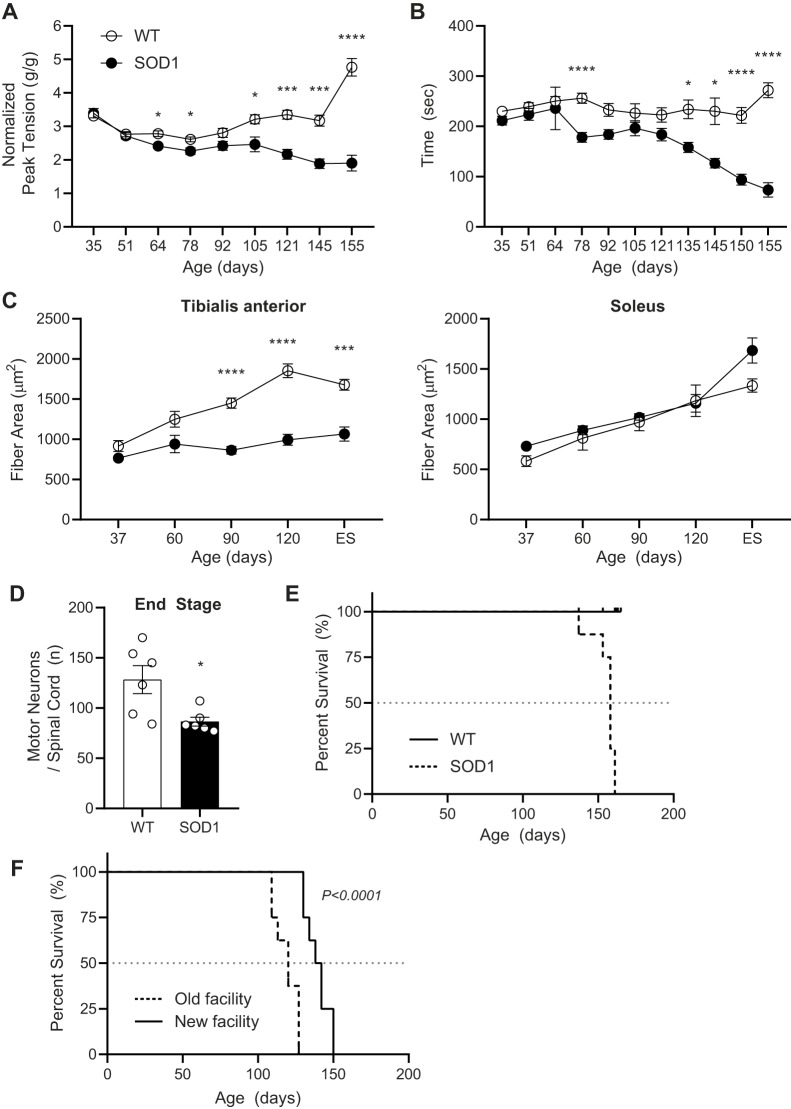
**Phenotypic changes in the ALS SOD1^G93A^ mouse model over time.** (A,B) Motor behavior was assessed by muscle strength by measuring grip strength (A) and motor coordination by the rotarod test (B). (C) Tibialis anterior (left panel) and soleus (right panel) muscle fiber cross-sectional areas were assessed in SOD1^G93A^ (black) and WT (white) mice at age 37, 60, 90 and 120 days and at end-stage (ES). (D) Loss of motor neuron density in the spinal cord of SOD1^G93A^ (black) versus WT (white) mice at 120 days and ES. (E) Decreased overall survival of SOD1^G93A^ (dashed black line) versus WT (solid line) mice is presented in a Kaplan–Meier curve with median survival indicated by horizontal dashed gray line. (F) Decreased overall survival of SOD1^G93A^ mice housed in old ‘dirty’ (dashed black line) versus new ‘clean’ (solid line) facility is presented in a Kaplan–Meier curve with median survival indicated by horizontal dashed gray line. For panels A-D: *P<0.01, ***P<0.001, ****P<0.0001, by two-tailed Student's *t*-test with multiple comparisons; *n*=8 mice per group per time point, except the 60-day-old ALS group for which *n*=7; data are mean±s.e.m. For panel E: *n*=8 mice per group. For panel F: *P*<0.0001, by Log-rank, Mantel-Cox test; *n*=8 mice per group.

To temporally map changes in the microbiome, immune response and epigenetic marks in relation to the onset and progression of neurodegeneration, we first assessed motor strength, motor coordination and muscle atrophy in SOD1^G93A^ and WT mice during disease progression (Fig. 1). As anticipated, transgenic SOD1^G93A^ mice exhibited a modest decline of muscle strength starting at ∼64 days of age (Fig. 2A), followed by worsening coordination that first becomes evident at 78 days of age, but was not consistently progressive until 135 days of age (Fig. 2B) (Henriques et al., 2010; Pfohl et al., 2015). In addition, the transgenic animals exhibited slight weight loss at ∼140 days, hind-limb tremors at ∼120 days, moderate paralysis in one limb, coat grooming changes and lethargy after ∼120 days (data not shown). Relative muscle atrophy, specifically in tibialis anterior (fast-contracting fibers) but not soleus (slow-contracting fibers) muscle tissue (Atkin et al., 2005; Pun et al., 2006) (Fig. 2C), was significantly different starting at 90 days of age when compared to WT animals. Tibialis anterior muscle fiber area was lower in SOD1^G93A^ mice aged 60 days, although this did not reach significance; however, it does align with the first signs of muscle weakness in forelimb grip strength at 64 days of age. Furthermore, the deterioration in neuromuscular function accelerated in the later stages of disease for all three metrics, which is mirrored by significant motor neuron loss in the spinal cord at end-stage (ES; Fig. 2D). The median survival of the transgenic animals was ∼150 days of age (Fig. 2E). Overall, these results confirmed that ALS developed as expected in our experimental mouse cohort and provided a timeframe for the onset and progression of disease based on motor behavior and neuromuscular decline for correlation with other disease metrics.

### SOD1^G93A^ mice exhibit altered gut microbiomes

The gut microbiome is implicated in several diseases, including ALS (McCombe et al., 2019). In serially housed mouse cohorts, we found that the survival of SOD1^G93A^ mice housed earlier in an older ‘dirty’ facility (*n*=8, median survival 120 days) was shorter than the survival of SOD1^G93A^ mice that were housed later in a newer ‘clean’ facility (*n*=8, median survival 140 days; *P*<0.0001; Fig. 2F). This retrospective observation suggested that the animals' intestinal flora influenced lifespan and that the gut microbiome in SOD1^G93A^ mice may experience temporal evolution over the course of ALS progression. In addition, we also wondered whether differences in gut microbiome existed between SOD1^G93A^ versus WT cohorts. To evaluate these possibilities more fully (i.e. temporal evolution and SOD1^G93A^ versus WT differences), we longitudinally profiled gut microbial communities in separate animal cohorts in fecal pellets and from terminal ileum and colon intestinal content from SOD1^G93A^ versus WT mice. The samples were submitted for 16S rRNA sequencing and analyzed to generate a map of microbial changes during ALS development in relation to the onset of other disease parameters. Most of the analysis of microbial diversity employed amplicon sequence variants (ASVs), which are unique sequences representing a detected microorganism. We also performed some analyses using operational taxonomic units (OTUs), which group highly similar sequences (≥97%) into clusters of microorganisms.

Intra-group gut microbiota diversity (i.e. degree of differentiation within microbial communities of a sample) was assessed by alpha diversity (richness, Simpson and Shannon indices) and inter-group diversity (i.e. degree of differentiation between microbial communities from different samples) was assessed by beta diversity of filtered ASVs. First, we examined all samples, both SOD1^G93A^ and WT together, stratified by location (colon, ileum, pellet; Fig. S1A,B) and over time (37, 60, 90 and 120 days; Fig. S1C,D). We observed overall that microbial alpha diversity in the ileum was lower compared to the colon and fecal pellets (Fig. S1A). Beta diversity also clustered the ileum distinctly from the colon and fecal pellets by principal coordinates analysis (PCoA; Fig. S1B). We observed higher alpha diversity earlier in the study in both the colon and ileum, with a general trend towards lower richness over time in the ileum, but a higher final diversity in the colon (Fig. S1C). In fecal pellets, alpha diversity generally increased over time. PCoA within all colon and ileum samples clustered ES samples together (Fig. S1D), again indicating evolution in microbial communities over time in both SOD1^G93A^ and WT mice.

**Fig. 3.**
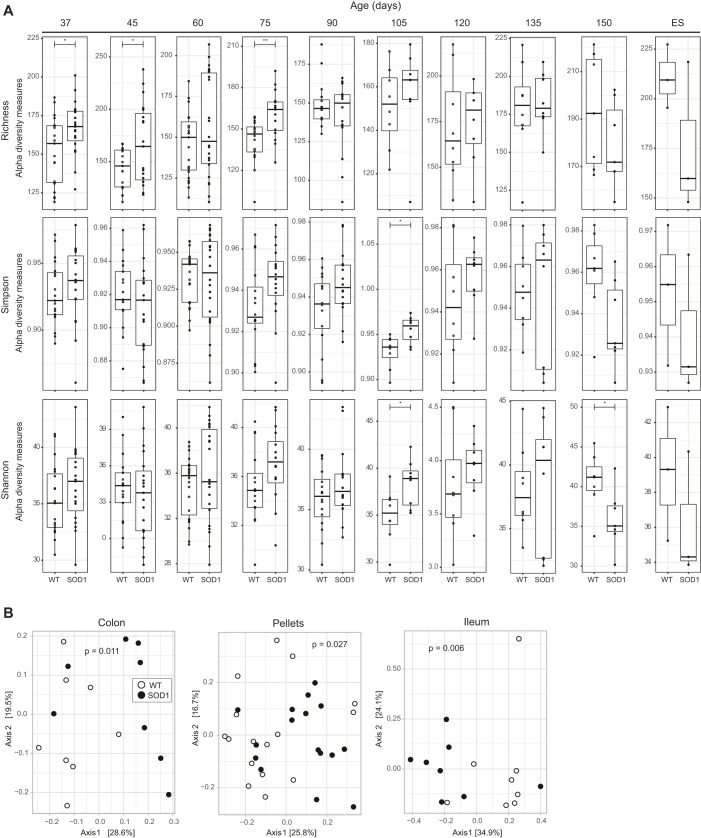
**The fecal pellet microbiome is altered in SOD1^G93A^ mice within a specific timeframe before disease onset.** (A) Alpha diversity by ASVs for fecal pellets collected every two weeks was determined by richness, Simpson and Shannon indices in SOD1^G93A^ (black) and WT (white) mice. Data are presented by box-and-whisker plots, with horizontal line the median, lower edge the first quartile, and upper edge the third quartile of each dataset and with whiskers indicating low and high values. *P<0.05, **P<0.01, by ANOVA. The number of animals (*n*) per group (SOD1^G93A^/WT) at each time point was: 37 days=19/19, 45 days=19/17, 60 days=22/23, 75 days=16/16, 90 days=16/16, 105 days=8/8, 120 days=8/8, 135 days=8/8, 150=5/5, ES=3/3. (B) PCoA plots representing beta diversity by ASVs of bacterial communities from colon, pellets and ileum samples of SOD1^G93A^ (black) and WT (white) mice aged 90 days. The number of animals (*n*) per group (SOD1^G93A^/WT) was: colon=8/8, *P*=0.011; fecal pellets=16/16, *P*=0.027; ileum=8/8, *P*=0.006. *P*-values by Adonis.

When we stratified alpha and beta diversity by SOD1^G93A^ versus WT mice, differences arose in intestinal flora among the animal cohorts over time. Alpha diversity of fecal pellets from SOD1^G93A^ mice was significantly higher between 37 and 105 days of age compared to WT mice but showed a relative, though nonsignificant, decrease at ES (Fig. 3A). On the other hand, no significant differences in alpha diversity were observed between experimental and control animals at any time point for colon and ileum samples (Fig. S2). Beta diversity was assessed by both phylogenetic (UniFrac) and non-phylogenetic (Euclidean, Bray-Curtis) methods and was considered significant if at least two methods produced a *P*-value lower than 0.05 by Adonis [permutational multivariate analysis of variance (MANOVA)]. For fecal pellets, significant differences between SOD1^G93A^ and WT mice were observed at age 60 and 90 days (Table S1). For intestinal bacterial content, we observed significant differences between SOD1^G93A^ and WT mice aged 90 days for both the colon and the ileum. Beta diversity analysis using OTUs instead of ASVs produced similar results, with significant differences uncovered by analysis of molecular variance (AMOVA, θ_YC_ distances) in fecal pellets between SOD1^G93A^ and WT at time points 60, 75 and 90 days and for the colon and the ileum at 90 days (Table S2). PCoA analysis demonstrated that the most distinct clustering of SOD1^G93A^ versus WT mice across all samples occurred in animals aged 90 days (colon, *P*=0.011; fecal pellets, *P*=0.027; ileum, *P*=0.006; Fig. 3B).

**Fig. 4.**
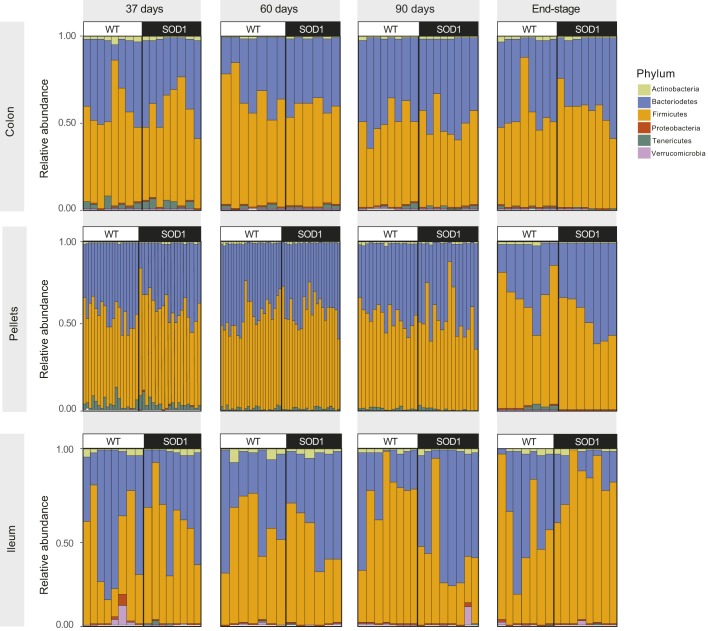
**Relative abundance of the most prevalent bacterial phyla in SOD1^G93A^ versus WT mice.** Distribution of Actinobacteria, Bacteroidetes, Firmicutes, Proteobacteria, Tenericutes and Verrucomicrobia by ASVs in colon, ileum and fecal pellets from SOD1^G93A^ and WT mice at 37, 60 and 90 days and at ES. Pairwise comparison between samples from SOD1^G93A^ versus WT mice was performed using a Mann–Whitney test for each F and B species that contributed >1% to abundance to evaluate *P*-values (Table S3). Number of animals was the same as in Fig. 3 for pellets and in Fig. S2 for colon and ileum samples.

ASVs were used to classify bacteria into the major phyla in SOD1^G93A^ and WT cohorts, among them Firmicutes (F) and Bacteroidetes (B), the two predominant phyla inhabiting the mouse gut (Wang et al., 2019), in addition to Actinobacteria, Proteobacteria, Tenericutes and Verrucomicrobia (Fig. 4). All F and B species that were detected in ileum, colon and fecal samples were considered for generating a net percentage of F and B within each sample (ileum, colon, pellet) and per mouse from both SOD1^G93A^ and WT groups (Fig. S3). We saw variability in relative F and B contributions across sample type (ileum, colon, pellet), between mice within the same cohort and between mice from different cohorts. We next performed a pairwise comparison between samples from averaged SOD1^G93A^ versus WT mice using a Mann–Whitney test for each F and B species that contributed >1% to abundance, to evaluate *P*-values and identify statistically significant differences in F and B contributions (Table S3). In the ileum, F were more abundant than B in SOD1^G93A^ (F=80.51%, B=17.18%) versus WT mice (F=56.12%, B=41.42%) at ES (F, *P*=0.05; B, *P*=0.03). The opposite was observed at 90 days, F being less abundant than B in SOD1^G93A^ (F=38.77%, B=57.73%) versus WT mice (F=71.63%, B=26.04%), although the difference was not significant (F, *P*=0.57; B, *P*=0.18). In fecal pellets, F was more abundant in SOD1^G93A^ cohorts in samples collected from mice aged 37 and 60 days (57.64 and 58.05%, respectively), in comparison to WT (52.52 and 55.06%, respectively) (37 days, *P*=0.02; 60 days, *P*=0.04). Finally, B species were more abundant in colon from the SOD1^G93A^ group (39.91%) than in the WT one (33.07%; *P*=0.02) in 60-day-old mice.

**Fig. 5.**
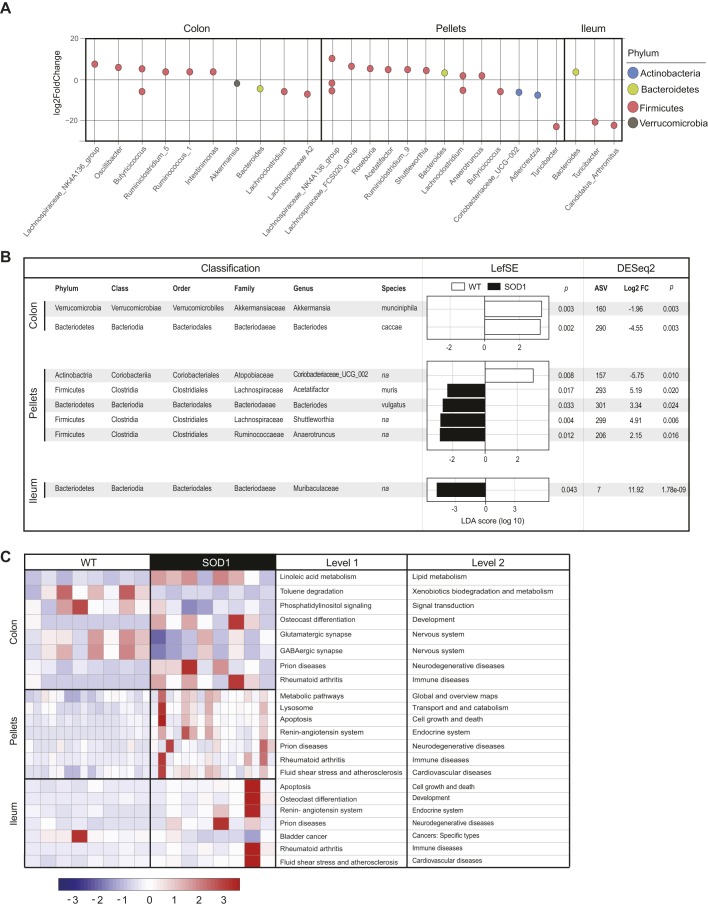
**Microbial communities in the gut are altered during early stages of ALS resulting in metabolic dysfunction.** (A) Differential abundance by ASVs in colon, fecal pellets and ileum expressed as Log_2_FC in SOD1^G93A^ versus WT animals aged 90 days. Each point represents an ASV at the genus level, *P-*value<0.05. (B) Combined LEfSe and DESeq2 of ASVs identifies the most highly differential species expressed as Log_2_FC in colon, fecal pellets and ileum in SOD1^G93A^ versus WT animals aged 90 days. (C) Representative heatmap of significant KEGG pathways (*y*-axis) associated with relative bacterial abundance in colon, fecal pellets and ileum in SOD1^G93A^ versus WT animals aged 90 days (*x*-axis, *n*=8 for colon and ileum, *n*=16 for pellets per group). The values are scaled by rows. FC, fold change.

After looking at net F and B contributions, we next used ASVs to consider only the bacterial genera that were associated with the greatest differences driving microbial diversity between the SOD1^G93A^ and WT mice aged 90 days, as that was the time point that beta diversity was greatest. In the colon and fecal pellets of 90-day-old mice, most ASVs that were significantly more abundant in SOD1^G93A^ mice compared to the WT group belonged to F, with the exception of those belonging to the genera *A2*, *Butyricicoccus*, *Lachnoclostridium* and *Turicibacter*, which were significantly less prevalent in SOD1^G93A^ mice (Fig. 5A). In the ileum of similarly aged mice, the F genera *Turicibacter* and *Candidatus Arthromitus* were reduced in the SOD1^G93A^ group. Of the phylum B, differences in microbiome could be ascribed to the genus *Bacteroides*, which was lower in colon but higher in ileum and pellets. Finally, the genus *Akkermansia* (phylum Verrucomicrobia) was diminished in the colon, and the Coriobacteriaceae and *Adlercreutzia* (phylum Actinobacteria) were lower in the pellets from SOD1^G93A^ mice in comparison to WT littermates. Next, we examined differential bacterial levels down to the species classification by linear discriminant analysis (LDA) effect size (LEfSe) analysis (species level, LDA>2) and DESeq2 analysis. Candidates that were common across both methods were considered as the species driving the microbial distinction between SOD1^G93A^ and WT mice. *Akkermansia muciniphila* and *Bacteroides caccae* were more prevalent in the colon of WT littermates compared to SOD1^G93A^ mice (Fig. 5B). In fecal pellets, two bacteria were identified down to the species level, *Acetatifactor muris* and *Bacteriodes vulgatus*, which were present in lower amounts in SOD1^G93A^ mice.

To infer functional biological pathways associated with the disruption in microbial communities, we performed Kyoto Encyclopedia of Genes and Genomes (KEGG) analysis of colon, ileum and fecal pellet samples. At 90 days of age, the most striking difference observed was upregulation in SOD1^G93A^ versus WT animals in KEGG pathways predicting functions related to neurodegeneration and immune diseases in all samples (Fig. 5C). Moreover, nervous system, lipid metabolism, xenobiotics biodegradation, development and signal transduction pathways were distinct between the two animal groups in the colon. Pathways related to the endocrine system, cardiovascular diseases, and cell growth and death overlapped in both pellets and ileum samples and were upregulated in SOD1^G93A^ mice.

### SOD1^G93A^ mice exhibit peripheral leukocyte changes and leukocyte infiltration into CNS tissue

**Fig. 6.**
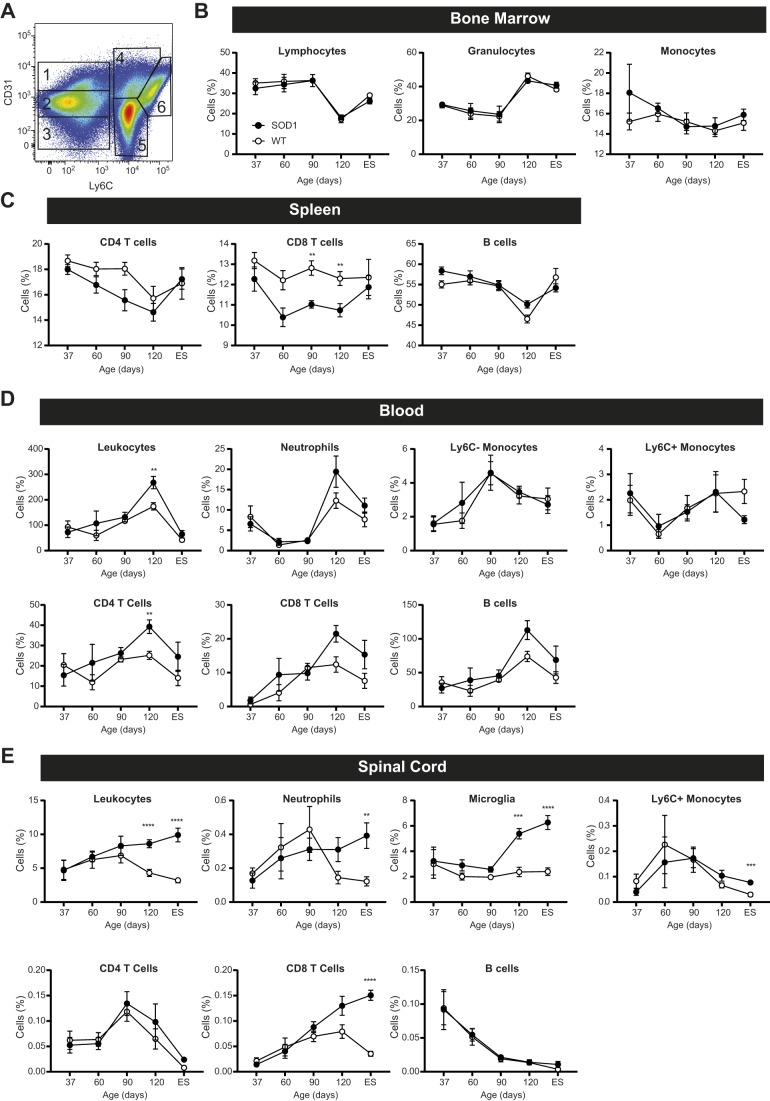
**Peripheral immune cell populations are altered during early ALS, and CNS inflammation occurs during late-stage disease.** (A-E) Immune cell populations were assessed in SOD1^G93A^ (black) and WT (white) mice at age 37, 60, 90 and 120 days and at ES. (A) Gating strategy for immune cell identification in bone marrow by flow cytometry using CD31 and Ly6C staining: (1) blast cells, (2) lymphocytes, (3) residual red blood cells, (4) transitioning cells, (5) granulocytes and (6) monocytes. Immune cell populations in bone marrow (B), spleen (C), blood (D) and spinal cord (E). **P<0.005, ***P<0.001, ****P<0.0001, by two-tailed Student's *t*-test with multiple comparisons. For bone marrow and spleen: *n*=8 mice per group at each time point, except the 60-day-old group for which *n*=7. The number of animals (*n*) per group (SOD1^G93A^/WT) was: blood, 37 days=8/6, 60 days=6/8, 90 days=8/8, 120 days=8/8, ES=8/8; spinal cord, 37 days=8/8, 60 days=7/8, 90 days=8/8, 120 days=8/8, ES=8/7; data are mean±s.e.m.

Next, we tracked the expansion and accumulation of immune cell populations during ALS progression in SOD1^G93A^ versus WT littermates aged 37, 60, 90 and 120 days and at ES in bone marrow, spleen, blood, brain and spinal cord tissue (Fig. 6). Bone marrow cells were analyzed by flow cytometry using CD31 and Ly6C staining (Murdock et al., 2012) to distinguish six distinct groups (Fig. 6A): progenitor blast cells (Group 1; CD31^high^ Ly6C^–^), lymphocytes (Group 2; CD31^mid^ Ly6C^–^), residual red blood cells (Group 3; CD31^low^ Ly6C^–^), transitioning progenitor immune cells (Group 4; CD31^high^ Ly6C^+^), granulocytes (Group 5; CD31^low^ Ly6C^+^), and monocytes and macrophages (Group 6; CD31^mid^ Ly6C^high^) (Nikolic et al., 2003; van der Loo et al., 1995). In other tissue, total immune cells were analyzed via CD45 expression (Figs S4 and S5). Within this group, lymphocytes were evaluated for a combination of CD3, CD4, CD8 and CD19 expression to identify CD4T cells (CD3^+^ CD4^+^ CD8^–^), CD8T cells (CD3^+^ CD4^–^ CD8^+^) or B cells (CD3^–^ CD19^+^). Myeloid cells were identified using side scatter (SSC), CD11b, Ly6C, Ly6G and CD45; we examined neutrophils (SSC^high^ CD11b^+^ Ly6G^+^), monocytes (CD45^high^ CD11b^+^ Ly6G^–^, Ly6C^±^) and microglia (CD45^mid^ CD11b^+^ Ly6G^–^).

Modest changes in leukocyte production were detected over time in the bone marrow but were not specific to SOD1^G93A^ mice (Fig. 6B). Our assessment of immune cell populations in the spleen was determined as a percentage of cells rather than as total number because SOD1^G93A^ mice tend to have undersized spleens and lose weight as the disease progresses (Banerjee et al., 2008). In SOD1^G93A^ mice, there was a trend towards reduced CD4T cell fraction at 90 days of age (*P*=0.023) and a significant decrease in the percentage of CD8T cells at 90 and 120 days of age, but no significant differences in B cell levels (Fig. 6C). Blood leukocytes were examined next for total cells and myeloid and lymphoid populations at all five time points per volume of blood. Total leukocytes and CD4T cells were significantly increased in ALS SOD1^G93A^ mice aged 120 days compared to WT controls, but trending with neutrophils (*P*=0.120), CD8T cells (*P*=0.017) and B cells (*P*=0.028) (Fig. 6D). We also observed a nearly 50% decrease in the number of pro-inflammatory monocytes (Ly6C^+^) in peripheral blood in mice aged 120 days until ES, although the differences did not reach significance after accounting for multiple comparisons (*P*=0.045).

Finally, we assessed total leukocytes as well as myeloid and lymphoid immune cells within the CNS, in both the brain and spinal cord. In the brain, we observed no significant immune differences at any of the five time points (Fig. S6). In contrast, we observed significant immune changes within the spinal cord in the later stages of disease (Fig. 6E). Increased total leukocytes were detected in ALS SOD1^G93A^ mice aged 120 days and at ES. Microglia were similarly elevated in 120-day-old SOD1^G93A^ mice and at ES, with trending towards increased numbers in 60-day-old (*P*=0.101) and 90-day-old (*P*=0.049) mice. Neutrophil and CD8T cell levels were also higher in SOD1^G93A^ mice at ES, with trends towards increased levels in 120-day-old mice (neutrophils: *P*=0.058; CD8: *P*=0.044). In addition to distinct immune cell levels in SOD1^G93A^ versus control spinal cord, the dynamics were also distinct. The infiltration of leukocytes, microglia, CD8T cells and neutrophils into SOD1^G93A^ spinal cord steadily increased over time, whereas levels first rose and fell in control spinal cord tissue. Ly6C^+^ monocytes were also higher in SOD1^G93A^ mice spinal cord at ES, although their levels fluctuated over time and followed the same pattern in both ALS and healthy spinal cord. There were no statistically significant differences in B cell levels. Together, these results show that there are marked alterations in immune cell phenotypes in the spinal cord of SOD1^G93A^ mice, particularly in the later stages of disease and in microglial, CD8T cell and neutrophil populations.

### SOD1^G93A^ mice exhibit increased myeloid activation and maturation in the CNS

Next, we assessed changes in activated microglia and Ly6C^+^ monocyte populations, as both have been implicated in ALS progression (Butovsky et al., 2012; Xiao et al., 2007). Activated immune cells were identified by flow cytometry via forward scatter (FSC, change in activation-induced cell size) and increased F4/80 and CD11c expression (Fig. S7) (Town et al., 2005; Greter et al., 2015). Consistent with total cell counts, there were very few differences in the brain between ALS SOD1^G93A^ and WT mice (Fig. S7A). Brain microglia from SOD1^G93A^ mice expressed slightly, yet significantly, lower CD11c at 60 and 90 days of age, with a trend towards increased expression at ES (*P*=0.010). However, no differences in FSC or F4/80 were noted, suggesting minimal differences in overall microglial activation in the brain. Similarly, SOD1^G93A^ and WT brain Ly6C^+^ monocytes did not exhibit differences in FSC and F4/80 expression, and CD11c expression was not detectable. Conversely, dramatic differences were seen in the spinal cord microglia of ALS SOD1^G93A^ mice at 120 days and ES (Fig. S7B), specifically a significant increase in microglial size and F4/80 and CD11c expression. In spinal cord Ly6C^+^ monocytes, there were no significant differences in SOD1^G93A^ versus WT mice, although a trend towards increased CD11c expression was noted in ALS SOD1^G93A^ spinal cord at ES (*P*=0.061). Cumulatively, these results indicate that spinal cord microglia are progressively activated as ALS develops.

### SOD1^G93A^ mice exhibit altered global brain 5hmC levels

Epigenetic marks, such as 5mC and 5hmC at cytosines, are altered during aging and neurodegeneration (Luo et al., 2018; Klein and De Jager, 2016). We assessed the global changes in these epigenetic marks in the CNS at study commencement (mice aged 37 days) and ES, and in ileum at ES. For the CNS, only brain tissue was available, as the immunophenotyping required all available spinal cord tissue. We observed an approximately 2-fold increase in global brain 5hmC in SOD1^G93A^ (2.58±0.33%; mean±s.e.m.) compared to WT mice (1.34±0.39%) at ES but not at the earlier time point (Fig. S8). In contrast, no significant changes in global 5hmC were observed in the ileum. Similarly, differences in global 5mC between SOD1^G93A^ and WT mice were not detected in either brain or ileum during the study duration.

### Correlation of metrics of ALS disease progression in SOD1^G93A^ mice

**Fig. 7.**
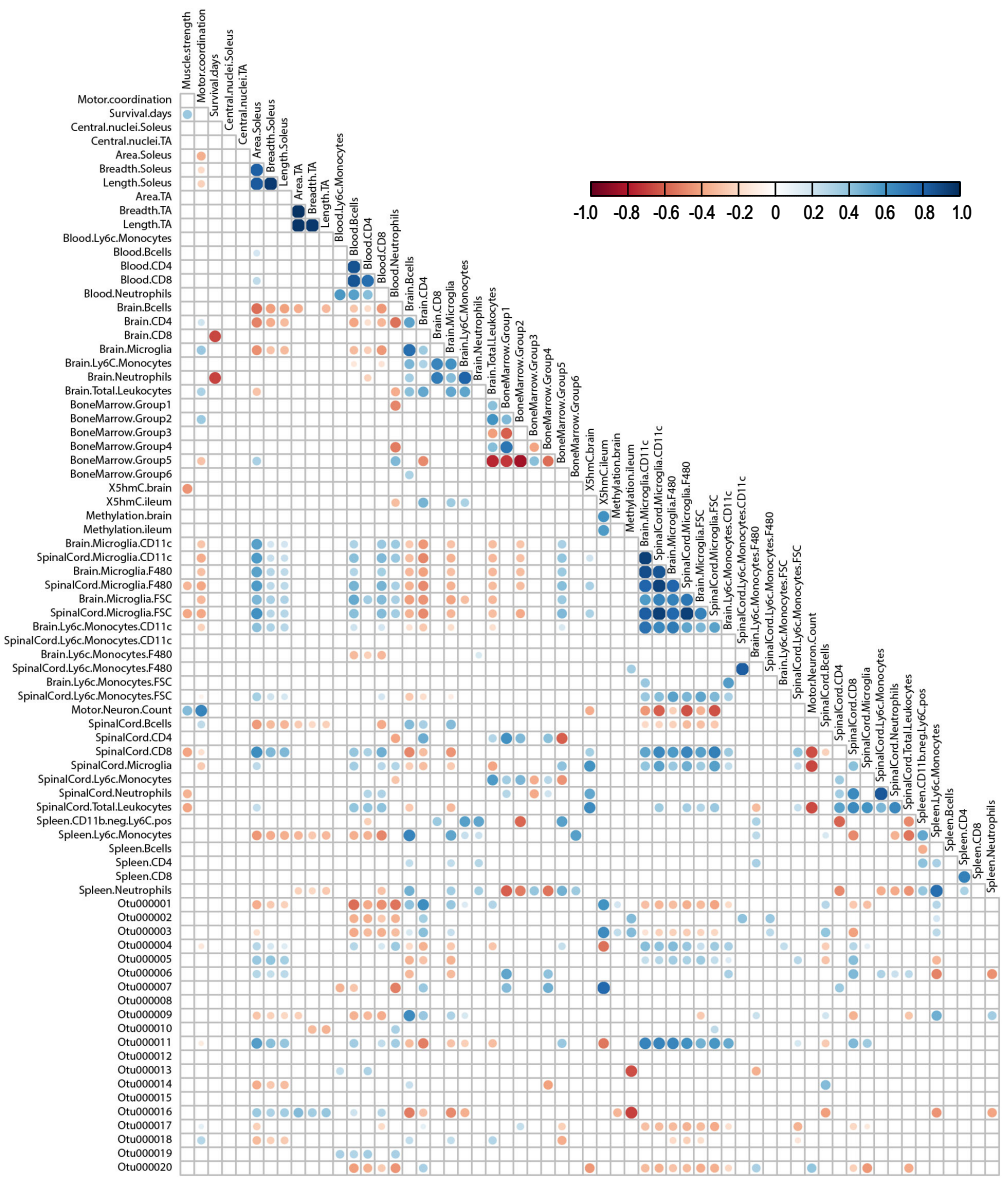
**Specific microbiome, epigenome and immune changes are correlated in ALS.** The correlation for the top 20 OTUs with immunophenotype, neuromuscular status and DNA methylation is shown. The analysis was performed using Pearson correlation. Significant correlations are plotted as a circle at the intersection between correlated parameters along the *y*-axis with parameters on the diagonal. Circles are color coded on a scale of highly positively correlated (1, blue) and highly negatively correlated (-1, red).

We finally determined whether the biological pathways we examined correlated, and possibly interacted, with one another, by performing global correlation analysis of the acquired data (i.e. microbiome, immunophenotype, 5mC/5hmC, muscle atrophy and motor coordination). We compared each measurement using Pearson correlation and plotted the significant associations, which we represented by circles plotted at the intersection of correlated parameters along the *y*-axis with parameters along the diagonal. Circles ranged from a strong positive (score of 1) to a strong negative (score of −1) correlation (Fig. 7, Fig. S9). OTUs were employed instead of ASVs to get a broader picture of microbiome correlations with other parameters. In addition, only the top 50 OTUs of 60,082 were considered in order to keep the number of potential correlations manageable (Table S4). The correlation displaying results with the top 20 OTUs is shown in Fig. 7 for clarity, whereas the full correlation displaying results from all 50 analyzed OTUs is shown in Fig. S9. Several anticipated positive correlations emerged, such as muscle fiber area to fiber breadth and length, as well as known positive correlations, for example spinal cord motor neuron density with motor coordination (Gurney et al., 1994).

The correlation analysis revealed potential associations between numerous pathological processes (i.e. motor neuron density, muscle atrophy, motor coordination, survival) with immunological, bacterial and epigenetic metrics. For example, motor neuron density negatively correlated with leukocyte and CD8T cell spinal cord infiltration and with immune cell activation, particularly in spinal cord and, to a lesser extent, brain microglia. Motor neuron density positively correlated with motor coordination and muscle strength, as expected, but also unexpectedly with intestinal colonization. Tibialis anterior muscle size (area, breadth, length) also correlated positively with intestinal colonization by certain bacteria.

The relative abundance of various bacteria was associated with distinct pathological processes in ALS (Table S5). Lachnospiraceae (phylum Firmicutes; OTUs 000021, 000023, 000025, 000027, 000031), Porphyromonadaceae (phylum Bacteroidetes; OTUs 000001, 000020) and Coriobacteriaceae (phylum Actinobacteria; OTU 000047) had among the strongest negative correlation with activated brain and spinal cord microglia. Lachnospiraceae (OTU 000025) in particular more strongly negatively correlated with spinal cord versus brain microglia. In contrast, Porphyromonadaceae (OTUs 000004, 000048), Erysipelotrichaceae (phylum Firmicutes; OTUs 000005, 000035), Lachnospiraceae (OTU 000038), and particularly Porphyromonadaceae (OTU 000011) correlated positively with microglial brain and spinal cord activation. All correlations with microglial activation similarly associated with spinal cord levels of CD8T cells, which are elevated in SOD1^G93A^ mice (Fig. 6E).

Global ileum 5hmC correlated positively with Porphyromonadaceae (OTUs 000001, 000003, 000007) and negatively with other Porphyromonadaceae family members (OTUs 000004, 000011). Global brain 5hmC associated inversely with only Ruminococcaceae (OTU 000037) with no strong positive correlations. Gut bacterial communities Ruminococcaceae (OTUs 000013, 000044) and Lachnospiraceae (OTUs 000016, 000045) also correlated negatively with ileum 5mC and positively, but less strongly, with Lactobacillaceae (OTU 000002) and Porphyromonadaceae (OTU 000003). No strong correlations emerged with brain 5mC levels.

## DISCUSSION

**Fig. 8.**
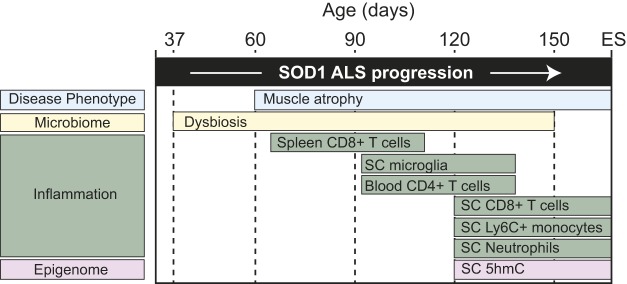
**Timeline of pathological events occurring during ALS progression in SOD1^G93A^ mice.** Evolution and potential interrelation of gut microbial, immune system and epigenetic changes in SOD1^G93A^ mice during progressive development of disease symptoms, i.e. loss of muscle fiber and strength and impaired coordination. SC, spinal cord.

We report a longitudinal study of three biological parameters with a potential role in ALS (i.e. gut microbiome, immune system, epigenome) relative to neuromuscular degeneration (i.e. muscle atrophy, grip strength, motor coordination) in an ALS SOD1^G93A^ mouse model. Our results indicate that significant differences in the microbiome of SOD1^G93A^ mice are evident beginning in mice aged 37 days (Fig. 3), suggesting that they precede the onset of deficits in tibialis anterior muscle fiber area (90 days), grip strength (64 days) and motor coordination (78 days; Fig. 2). In the presence of these shifts in microbial communities and neuromuscular deterioration, the immune landscape also changes. Moderate differences in ALS SOD1^G93A^ mice versus WT arise in the peripheral system beginning at 90 days, with elevated CD8T cells in the spleen (Fig. 6C), and then at 120 days with CD4T cell counts in peripheral blood (Fig. 6D). However, the most profound changes occur with a steady expansion of microglia, CD8T cells and neutrophils in ALS SOD1^G93A^ spinal cord, which becomes statistically significant versus WT starting at 120 days (Fig. 6E). Furthermore, the spinal cord microglia adopt an activated phenotype, with increased size and F4/80 and CD11c expression (Fig. S7). Finally, comparison of brain cytosine methylation reveals increased SOD1^G93A^ brain 5hmC levels relative to WT at ES (Fig. S8). Overall, our results provide a roadmap to the chronological changes that occur in the gut microbiome and immune system relative to disease onset and progression in the SOD1^G93A^ mouse model (Fig. 8).

Our cohort of SOD1^G93A^ mice developed ALS, with the first signs of symptom onset occurring at 64 days for grip strength, 78 days for rotarod motor coordination and 90 days for muscle atrophy (Fig. 2A-C). Our ALS cohort exhibited onset relatively early for this genetic background compared to the literature, but as anticipated, definitive and progressive decline began at 105 days (Pfohl et al., 2015). Furthermore, this strain of mutant SOD1 mice does not display significant sex differences in motor dysfunction and survival, so we chose to employ both male and female mice more akin to the approximate 1:1 ratio in human ALS patients with mutant SOD1 (Orrell et al., 1999).

Microbiome analysis in all pooled samples (i.e. both SOD1^G93A^ and WT) stratified by tissue revealed lower overall diversity in ileum compared to colon and fecal pellets (Fig. S1A,B). The ileum contains fewer bacteria than the colon (Sekirov et al., 2010) and is less phylogenetically diverse in the two predominant phyla, F and B (Frank et al., 2007), which could explain the lower overall diversity we found in the ileum. The role of the gut microbiome on human health and disease is increasingly appreciated (Lynch and Pedersen, 2016), including as a possible factor in ALS (McCombe et al., 2019). Indeed, when we compared SOD1^G93A^ to WT mice, we observed significant differences in the gut microbiome beginning early, already at age 37 days, and spanning over a period until 105 days. Specifically, we found microbial alpha diversity in fecal pellets was significantly greater in SOD1^G93A^ compared to WT mice (Fig. 3A), a trend that was reversed, albeit not significantly, after 150 days. When considering beta diversity, the greatest separation of SOD1^G93A^ versus WT clusters upon PCoA occurred at 90 days (Fig. 3B).

The relationship between microbial diversity and healthy aging in WT animals versus aging in SOD1^G93A^ animals is complex. Although high diversity during aging is generally linked to a healthy microbiome, some studies suggest that it is the gain or loss of specific bacterial species, or ‘core microbiota’ that drives aging or disease and not diversity per se (Jeffery et al., 2016). Although alpha diversity was distinct in SOD1^G93A^ relative to WT fecal pellets, we saw no significant differences in diversity in ileum or colon content (Fig. S2). This may arise because of distinct bacterial populations in expelled fecal pellets, which are an amalgamation of bacteria prone to shedding from the mucosa, versus ileum and colon samples that comprise proportionately more non-shedding mucosal bacteria (Eckburg et al., 2005).

To look beyond diversity, we identified the genera that accounted for the differences seen between SOD1^G93A^ and healthy mice. Differential ASVs classified microorganisms into six major phyla: F, B, as the two most prevalent, along with Actinobacteria, Proteobacteria, Tenericutes and Verrucomicrobia (Fig. 4). When considering the F and B contributions in SOD1^G93A^ mice, F were significantly more abundant than B in ileum samples from SOD1^G93A^ versus WT mice at ES, with an opposite trend at 90 days, though this only approached statistical significance (Fig. S3, Table S3). F was similarly more abundant in SOD1^G93A^ compared to WT cohorts in fecal pellet samples collected from mice aged 37 and 60 days, whereas B species were more abundant in colon from 60-day-old SOD1^G93A^ versus WT mice. Differences in B (and hence in F and B contributions) in SOD1^G93A^ versus WT were mostly ascribed to the genus *Bacteroides*, whereas several F genera differed (Fig. 5A). Other studies in the same SOD1^G93A^ mouse found lower butyrate-producing bacterial flora (F) and similar B levels in fecal/cecal samples, also suggestive of a lower F/B ratio (lower relative F, higher relative B contributions) in SOD1^G93A^ mice (Wu et al., 2015; Zhang et al., 2017), but evolution over time was not investigated. Small pilot studies in human ALS patients report lower F/B ratios in fecal samples (Rowin et al., 2017; Fang et al., 2016), but larger studies do not replicate these findings (Brenner et al., 2018). Differences among studies may be due to patient selection, as some patients had gastrointestinal (GI) problems (Rowin et al., 2017), a common complication in ALS (Toepfer et al., 1999), whereas other studies purposely excluded patients with GI complaints (Brenner et al., 2018). Moreover, our studies in mice show that F and B contributions (and hence the F/B ratio) evolve over time; therefore, our data suggest that examining the microbiome at only one time point and not accounting for the stage of ALS may be a factor for the differing results in F/B ratios reported in human ALS patients.

F and B contributions (F/B ratio) is a broad classification; however, specific species may be responsible for disease phenotype. Therefore, we examined differences in bacteria down to the species level (Fig. 5B). Several candidates emerged in colon (*Akkermansia muciniphila* and *Bacteroides caccae*) and fecal pellets (*Acetatifactor muris* and *Bacteriodes vulgatus*); in each case these species were less prevalent in SOD1^G93A^ mice versus WT littermates. Our results agree with a recent report that low levels of *Akkermansia muciniphila* are present in SOD1^G93A^ animals, which plays a role in disease severity. In a study by Blacher et al., colonization of germ-free SOD1^G93A^ mice with *Akkermansia muciniphila* slowed disease progression and improved animal survival (Blacher et al., 2019). Analysis of human ALS fecal samples by the same investigators did not recapitulate the relatively lower abundance of *Akkermansia muciniphila* but did find lower levels of its metabolite nicotinamide in serum and cerebrospinal fluid. In contrast to our findings of lowered levels of *Bacteriodes vulgatus*, Rowin et al. identified increased levels of *Bacteroides vulgatus* in stool samples from two ALS patients (Rowin et al., 2017). This could be because of the small sample size or an inherent difference between the ALS mouse and human patients. Interestingly, although a lower F/B and higher B is generally associated with ALS (Rowin et al., 2017; Fang et al., 2016; Wu et al., 2015; Zhang et al., 2017), two species belonging to B, *Bacteroides caccae* and *Bacteroides vulgatus*, were lower in our cohort of SOD1^G93A^ mice. These data suggest that relying solely on F and B contributions (i.e. phyla) may be misleading, as species-specific microbes may be more important factors in ALS progression (Blacher et al., 2019).

Gut bacteria outnumber cells of the human body, and cumulatively constitute ∼500-1000 species that flood the host intestine with metabolites and signaling molecules that impact biological processes (Sekirov et al., 2010). Although we did not perform metagenomic shotgun sequencing, which is the most reliable method of functional profiling of bacterial communities, we employed a computational program, Tax4Fun2, which inferred biological function from ASVs. KEGG analysis of these predicted functional processes in SOD1^G93A^ against WT microbiome can highlight important pathways that are dysregulated in ALS (Fig. 5C). At KEGG level 2, pathways related to neurodegenerative (prions) and immune diseases were generally upregulated in all samples (colon and ileum content, and fecal pellets). Association of ALS with aberrant immune system health and the gut microbiome has been suggested and is a recurrent theme in intestinal content and fecal samples from our cohort of SOD1^G93A^ animals and from similar ALS mouse studies (Pellegrini et al., 2018; Wu et al., 2015). Other affected pathways with possible points of convergence between microbiome and ALS include lipid metabolism (Ferri and Coccurello, 2017; Wang et al., 2016), xenobiotic biodegradation (toluene) (Ratner et al., 2018) and development (osteoclast differentiation) (Novince et al., 2017; Zhu et al., 2015), among others. Overall, analysis of intestinal flora in SOD1^G93A^ versus WT littermates suggests dysbiosis may be occurring in ALS.

Herein, we also found that there were differences in immune cell populations in SOD1^G93A^ versus WT mice, early within the spleen and peripheral blood, and within the spinal cord at disease ES (Fig. 6). Our results indicate that changes in the immune system may occur relatively early in ALS and may be progressing from the periphery to the CNS. Given the relatively mild level of inflammation in ALS (Keizman et al., 2009), it is perhaps unsurprising that we did not see large differences in the production of bone marrow progenitors. This, however, does not rule out the possibility that epigenetic changes are occurring in these cells that may impact their function (Figueroa-Romero et al., 2012). In contrast, reduced CD8 – and to a lesser extent CD4 – T cell levels in the spleen may account for subsequent increased leukocytes in the blood. Given the lack of changes in the bone marrow with regards to cellular production, release of T cells from the spleen may account for the reduced spleen size that has been previously observed in later stages of ALS (Banerjee et al., 2008).

In the current mouse study, we observed that CD4T cell levels increase in the peripheral blood during early to mid-stages of ALS but drop as the disease progresses. The lymphocyte kinetics we observed in peripheral blood may help resolve discrepancies between previously reported human studies. Whereas one study in man observed increased CD4 and CD8T cell levels (Gustafson et al., 2017), we previously reported a reduction in total CD4T cells in ALS patients (Murdock et al., 2016), which continued to decrease over time (Murdock et al., 2017). The patient cohort in the first study was examined during an earlier stage of ALS, whereas our patients were evaluated in the later stages of disease. Therefore, it is plausible that a similar temporal pattern occurs in human patients as we observed in SOD1^G93A^ mice, which may account for the differences reported between human studies.

In addition to changes in the periphery, we saw an expansion of certain immune cell populations in the spinal cord, including CD8T cells, microglia and neutrophils (Fig. 6E). Indeed, CD8T cell expansion in the spinal cord is consistent with the literature in mice (Chiu et al., 2009) and human ALS patients (Engelhardt et al., 1993; Fiala et al., 2010). Recently, a report demonstrated that CD8T cells in SOD1^G93A^ mice may be selectively destroying motor neurons in ALS (Coque et al., 2019). The study also found that CD8T cells infiltrated to the spinal cord at 90 days onwards but displayed a distinct pattern of surface marker expression compared to peripheral CD8T cells. In addition, the authors report that CD8T cell counts in peripheral blood did not differ significantly between SOD1^G93A^ and WT mice, as we also observed (Fig. 6D).

We also found changes in the spinal cord microglia population. Although expansion and activation of microglia have previously been established in ALS (Zhao et al., 2010; Henkel et al., 2006, 2004; Boillee et al., 2006), we found that spinal cord microglia from SOD1^G93A^ mice expressed higher levels of F4/80 and CD11c as the disease progressed (Fig. S7B), also consistent with previous observations (Chiu et al., 2008). These data suggest that peripheral monocytes may be trafficking into the CNS during disease development, as recent reports suggest that all F4/80- and CD11c-expressing microglia in immune-privileged tissue, such as the spinal cord, are derived from infiltrating peripheral Ly6C^+^ monocytes and not from the resident microglial pool (O'Koren et al., 2016). In addition, Ly6C^+^ monocyte accumulation has been implicated in ALS progression in mouse models (Butovsky et al., 2012), and we observed a trend toward fewer Ly6C^+^ monocytes in the blood during late disease, suggesting potential recruitment into the tissue. Ly6C^+^ monocytes have a turnover of ∼4 days under non-inflammatory conditions (Janssen et al., 2016), thus it is unlikely that cellular accumulation in the CNS alone could account for a significant drop in peripheral Ly6C^+^ monocyte levels. However, monocyte-derived macrophages have been shown to infiltrate other tissue during ALS, such as the peripheral nerves and skeletal muscle (Kano et al., 2012; Troost et al., 1992; Chiu et al., 2009). It is therefore possible that monocyte infiltration into multiple tissue types is responsible for the reduced levels of peripheral Ly6C^+^ monocytes observed in peripheral blood. Our observations provide further evidence that peripheral monocytes may influence the course of ALS and may serve as a therapeutic target (Butovsky et al., 2012; Gustafson et al., 2017; Vaknin et al., 2011).

Interestingly, we also found that neutrophil levels significantly increased in the spinal cord of SOD1^G93A^ mice at later stages of disease. Though previous studies have found increased levels of circulating neutrophils in human patients (Desport et al., 2001; Chio et al., 2014; Keizman et al., 2009; Murdock et al., 2016; Banerjee et al., 2008), this is one of few studies that demonstrates progressive neutrophil accumulation in the spinal cord during ALS development. These data are also consistent with our recent longitudinal study that found a highly significant correlation between increased blood neutrophil levels in patients with ALS progression (Murdock et al., 2017).

The epigenome represents a point of convergence between genetics and environmental factors, and suggests a mechanism that could affect ALS onset and progression (Paez-Colasante et al., 2015; Al-Mahdawi et al., 2014). Therefore, the last parameter we investigated was ileum and brain cytosine methylation, of both 5mC and 5hmC levels. Loci-specific differences in methylation are seen in ALS frontal cortex and cerebellar brain tissue (Ebbert et al., 2017), as well as increased global 5mC and DNA methyltransferase levels in ALS motor cortex (Chestnut et al., 2011). Therefore, we anticipated that differential cytosine methylation marks may be present in SOD1^G93A^ versus WT animal brain tissue. In addition, differential DNA methylation occurs in distinct sections of the gut in inflammatory bowel disease, advocating analysis of ileum tissue (Howell et al., 2018). We found that brain 5hmC at ES was significantly higher in SOD1^G93A^ versus WT mice (Fig. S8A), although there were no differences in 5mC levels (Fig. S8B). Also, no distinctions in either 5mC or 5hmC were noted in ileum tissue at ES (Fig. S8C,D). 5mC at gene promoters is known to silence gene expression, but the role of 5hmC is less established, although it has been proposed as an intermediate along the dynamic cytosine demethylation and methylation landscape and also as a modification in its own right (Al-Mahdawi et al., 2014). 5hmC generally occurs at a lower level than 5mC, and this is recapitulated in our results. Furthermore, in the substantia nigra of WT mice, 5hmC levels are elevated in old versus younger mice, with no differences noted in 5mC (Fasolino et al., 2017). With regards to ALS, most studies in patients have examined differences in DNA methylation in whole blood, including in patients with mutant SOD1, and found no differences in global methylation, although older epigenetic age was noted in ALS patients (Tarr et al., 2019).

Among the correlations our study uncovered (Fig. 7), tibialis anterior muscle size (area, width, length) in ALS correlated positively with Lachnospiraceae (F; OTU 000032). This agrees with emerging data on a potential role for the microbiome on muscle in aging (Ni Lochlainn et al., 2018), and this may be an aspect in ALS as well. The correlation analysis also revealed both potential positive and negative associations of the microbiome with spinal cord inflammation. Although this has not been investigated widely in the context of ALS, animal models and human studies of spinal cord injury reveal a possible dysbiosis or disease-modifying action of the microbiome on spinal cord inflammation post spinal cord injury (Kigerl et al., 2018). In a multiple sclerosis model, the presence of *Prevotella histicola* in the gut microbiome protects against autoimmunity and inhibits inflammation and neuron demyelination in the spinal cord (Mangalam et al., 2017). Collectively, our data and these cited studies suggest that intestinal flora can affect inflammatory outcomes in the spinal cord, which could influence neurodegenerative disease progression. In contrast to our results, the comprehensive microbiome study by Blacher et al. did not find differences in the immune system of SOD1^G93A^ animals (Blacher et al., 2019), although it is a well-established hallmark of both SOD1^G93A^ animals and ALS patients (Thonhoff et al., 2018; Chiu et al., 2008, 2009; Engelhardt et al., 1993; Fiala et al., 2010; Gustafson et al., 2017; Murdock et al., 2016, 2017; Alexianu et al., 2001; Coque et al., 2019).

The correlation analysis also revealed an association between global ileum 5mC and 5hmC levels in ALS with diverse gut microbes, and with global brain 5hmC with Ruminococcaceae. Bacteria can influence the epigenome by secreting metabolites and modulating the immune system (El Aidy et al., 2016; Qin and Wade, 2018), but this has not been specifically investigated in ALS. A correlation has been seen between metabolic syndrome-induced low-grade inflammation with altered loci-specific methylation and the microbiome, specifically lower *Faecalibacterium prausnitzii* of the Ruminococcaceae family, in type 2 diabetes and obese individuals versus lean controls (Remely et al., 2014). Overall, the correlation analysis pinpointed potential associations between the immune system, epigenome and microbiome as avenues of future research for ALS.

In summary, leveraging the microbiome and the immune system to develop novel mechanism-based ALS therapies is limited by the lack of detailed temporal maps relative to symptom onset and disease progression. In this study, we used a familial ALS SOD1^G93A^ mouse model to perform the most comprehensive longitudinal evaluation to date of chronological changes that occur in the microbiome, the immune response and epigenetic marks. The study had some limitations, namely, that the 120-day cohort was a separate cohort, which could introduce variability, and that the number of males to females was not always equal at each time point. In addition, the ‘dirty’ versus ‘clean’ vivaria were retrospective observations lacking experimental controls that were made serially rather than in tandem and only compromised eight animals per cohort. The non-paralleled setup precludes discussion of causality; however, these preliminary findings warrant further study. Indeed, manipulation of the gut microbiome in SOD1^G93A^ mice can affect survival (Blacher et al., 2019). Overall, our study represents a significant step towards defining the evolution in intestinal microbiota, circulating and CNS immune system expansion and activation, and global ileum and brain cytosine modifications in SOD1^G93A^ mice relative to symptom onset and progression. Our data establish a time line that may pinpoint biomarkers of ALS for earlier diagnosis and therapeutic intervention, such as changes in gut microbiome architecture, as well as support novel therapeutic avenues aimed at immune system modulation (Miller et al., 2014; Wosiski-Kuhn et al., 2019). In the future, manipulation of gut microorganisms and/or the immune system in ALS patients may offer a route for the discovery of early-stage therapeutic interventions, biomarkers and diagnostic techniques to improve survival outcomes.

## MATERIALS AND METHODS

### Study design and animal model

The study began with procurement of thirty-day-old male and female SOD1^G93A^ mice [B6.Cg-Tg(SOD1*G93A)1Gur/J (SOD1^G93A^); Stock No: 004435] and their WT control littermates (The Jackson Laboratory). Sex differences in disease onset and progression in SOD1^G93A^ mice on a C57BL/6 background are negligible (Pfohl et al., 2015), and we intended to examine a cohort more similar to a human patient population, which is approximately 1:1 male:female in familial ALS (fALS) with mutant SOD1 (Orrell et al., 1999). Upon arrival, animals were housed in a dedicated facility maintained at 20±2°C with a 12 h light/12 h dark cycle and provided water and 5L0D chow (LabDiet) *ad libitum*. Towards ES, when the mobility of SOD1^G93A^ mice was significantly reduced, they were additionally provided gel food (DietGel^®^ 76A, 72-07-5022, ClearH_2_O), which is more readily accessible than chow. Veterinary staff monitored the mice daily. Each animal was genotyped by PCR on tail genomic DNA using a protocol from The Jackson Laboratory. All experiments were performed in accordance with protocols approved at the University of Michigan by the Institutional Animal Care and Use Committee (IACUC) (approval #PRO00008431) and the National Institutes of Health’s Guide for the Care and Use of Laboratory Animals (8th Edition).

SOD1^G93A^ and WT mice were weighed weekly, whereas rotarod performance and grip strength were recorded at 35, 51, 64, 78, 91, 105, 121, 135*, 145, 150* and 155 days (*denotes dates that grip strength was not assessed; Fig. 1). SOD1^G93A^ and WT mice aged 37, 60, 90, 120 days and ES were sacrificed for tissue harvest (*n*=8 per time point, except for the 60-day-old ALS group, for which *n*=7), which was used for immunophenotyping (blood, spleen, brain, spinal cord), analysis of skeletal muscle atrophy, and microbiome analysis of ileum and colon content (ileum and colon samples were not collected at 120 days of age). Microbiome analysis of fecal pellets was performed at 37, 45, 60, 75, 90, 105, 120, 135 and 150 days and at ES on all mice still alive at that time point (i.e. *n* decreased as animals were harvested). Spinal cord tissue from SOD1^G93A^ and WT mice at ES was used to determine motor neuron density and for immunophenotyping. Brain tissue from SOD1^G93A^ and WT mice at study start (aged 37 days) and at ES was used for epigenetic analysis of global 5mC and 5hmC levels. Ileum tissue was used for 5mC and 5hmC assessment at ES. SOD1^G93A^ and WT mice were received from The Jackson Laboratory in two separate batches at two distinct times, which each contained both SOD1^G93A^ and WT mice. Experiments were performed on mice from both batches at all time points for a total of two biological replicates containing six to eight technical replicates per genotype per time point. The number of mice selected for the study design was based on earlier results (Paez-Colasante et al., 2013; Murdock et al., 2012, 2011, 2014a,b).

Mice were sacrificed by lethal pentobarbital (Vortech Pharmaceutical) injection before tissue harvest. All mice were part of the original two cohorts, with the exception of mice sacrificed at 120 days of age, which were part of a separate independent cohort. A final group of SOD1^G93A^ animals was allowed to reach disease ES (∼160 days) and sacrificed when they were no longer able to right themselves, a symptom used to humanely determine death was approaching. Control littermates were sacrificed in parallel at each ES time point. Lifespans of this final group were used to generate a Kaplan–Meier survival curve using Prism (GraphPad).

### Motor function analysis

Overall motor function in each mouse was assessed every 2 weeks using a rotarod series 8 instrument (IITC Life Science) as previously reported (Seaberg et al., 2015). Briefly, training started 4 days after the mice arrived, and the first measurement was recorded the day after when they were aged 35 days. Mice were first exposed to a constant speed of 4 rpm for 5 min. Next, the rotarod was accelerated at 0.1 rpm s^–1^ from a starting speed of 4 rpm, up to a maximum of 30 rpm for 5 min. The time to fall was recorded. There were 10-min rest periods between three total trials, which were used to calculate an average rotarod performance. Time to fall per mode was normalized to individual weight, which was recorded weekly. Forelimb grip strength was evaluated biweekly for each animal using a grip strength meter with a single sensor and a standard pull bar and software (Columbus Instruments) (Seaberg et al., 2015). The peak force normalized to body weight was recorded in three consecutive trials and used to report an average grip strength.

### Myofiber area analysis

Tibialis anterior and soleus muscles were collected during animal harvest and frozen, and transverse frozen sections were analyzed using dystrophin staining. In brief, tibialis anterior and soleus muscles were dissected in their entirety and flash frozen in Tissue-Tek^®^ O.C.T. Compound (Sakura^®^ Finetek) using a 50/50 ethanol/dry ice bath. Cross-sections (20 µm thick) were cut using a cryostat (Leica CM1850, Leica Biosystems) and tissue was mounted on slides. Sections were fixed in paraformaldehyde (PFA, 4%) for 10 min and then rinsed for 5 min three times in phosphate buffered saline (PBS) with Tween 20 (PBS-T). Tissues were blocked in 10% normal goat serum in PBS, incubated overnight with an anti-dystrophin antibody (1:300, Abcam, ab15277) followed by incubation with a fluorescein-conjugated goat anti-rabbit secondary antibody (1:1000, Thermo Fisher Scientific, A-11034) and mounted for imaging (Table S6). Images of myofibers spanning across ∼35% of the total section were taken at 10× using a Microphot-FX4 and SPOTRT color CCD camera (Nikon USA). Care was taken to obtain images of non-overlapping areas within each tissue section. Analysis of myofiber area was conducted using the MetaMorph software (version v7.1.2.0, Molecular Devices) as previously described (Paez-Colasante et al., 2013). The area from as many myofibers as possible per image was measured.

### Motor neuron count in spinal cord analysis

Cervical spinal cord tissue was collected in its entirety by careful dissection to preserve structure and morphology during animal harvest from ES mice. The segments were fixed in 4% PFA for 24 h and then cryoprotected in increasing concentrations of sucrose for 3 days. Spinal cords were embedded in O.C.T. medium, frozen in a 50/50 ethanol/dry ice bath and sectioned in a cryostat (12 µm thick). Tissues were briefly stained with toluidine blue, washed in PBS, mounted with ProLong® Gold Antifade Mountant (P36930, Invitrogen, Thermo Fisher Scientific) and imaged using an Olympus BX43 microscope (Olympus USA), first at 4× to obtain an image of the entire ventral half of the spinal cord, and then at 10× and 20× for higher magnification to identify motor neurons. Motor neurons from both ventral horns from one section per animal were manually counted by a blind observer.

### Microbiome analysis

#### Comparison of housing conditions on SOD1^G93A^ mouse lifespan

Survival of SOD1^G93A^ mice (*n*=8) was assessed in an old animal housing facility at Oxford University, which was considered to be relatively ‘dirty’ compared to modern-day facilities. Later, survival was evaluated in another cohort of SOD1^G93A^ mice (*n*=8), which were housed in a newly built, state-of-the-art animal housing facility that was considered ‘clean’. The old ‘dirty’ facility was maintained at 20±1°C with 45-55% humidity, and the air was changed 20 times per hour. It employed conventional caging (i.e. not individually ventilated; MB3, NKP Cages) that housed a maximum of eight animals per cage. Bedding and cages were cleaned weekly. The ‘dirty’ facility did not place restrictions on the equipment that was brought in nor on animal imports, which were usually accepted directly into existing holding rooms with few exceptions.

The new ‘clean’ facility was maintained at 20±1°C with 45-55% humidity, and the air was changed 15 times per hour. The fewer air changes were offset by employing individually ventilated cages (1145T, Tecniplast), which significantly reduced the risk of laboratory animal allergens to the operators. Cages in the ‘clean’ facility housed a maximum of five animals per cage, and bedding and cages were also cleaned weekly, except cleaning was performed by a dedicated service area team, and all cages and bedding were autoclaved. The ‘clean’ facility placed restrictions on the equipment that was brought in, which had to be thoroughly cleaned and sterilized by vaporized hydrogen peroxide before admittance into the facility. The ‘clean’ facility also only directly accepted animals from commercial breeders, otherwise animals had to be quarantined until their health status could be confirmed by rigorous testing. Both old ‘dirty’ and new ‘clean’ facilities required sterile aseptic techniques and full personal protective equipment for all surgical procedures. The survival data were plotted on a Kaplan–Meier curve and statistical significance was determined with log-rank, Mantel-Cox test using Prism (version 7.01, GraphPad).

#### Fecal and intestinal content sample collection

Expelled fecal pellets were collected directly from animals into an Eppendorf tube before tissue harvest at the first time point (37 days), when aged 45 days, and every 2 weeks after until the experimental endpoint. Intestinal content from terminal ileum and colon were obtained at 37, 60 and 90 days and at ES during tissue harvest by excising ∼3 mm of intestine (either ileum or colon) under sterile conditions. All collected samples were stored in PowerMicrobiome™ RNA Isolation Kit, Glass Bead Tubes, 0.1 mm (MO BIO Laboratories) and stored at −80°C until bacterial DNA extraction.

#### Isolation of bacterial DNA and sequencing

Bacterial DNA was isolated from intestinal content and fecal samples using a PowerMag® Microbiome RNA/DNA Isolation Kit (MO BIO Laboratories) on an epMotion® robot (Eppendorf). DNA sequencing of the V4 region of the bacterial 16S rRNA gene was performed on a MiSeq instrument (Illumina) in paired-end, ∼250 bp format at the University of Michigan Microbial Systems Molecular Biology Laboratories as previously described (Seekatz et al., 2015).

#### Data analysis for ASVs

The raw FASTQ files were filtered with the dada2 package by using filterAndTrim on sequences that were truncated to a length for both paired-end reads [truncLen=c(150,130)] (Callahan et al., 2016). Reads were discarded if they had more than two expected sequencing errors (maxEE=2). Filtered sequences were then dereplicated using the dada2 function derepFastq, generating ∼70,766 unique sequences per sample. The paired-end reads were joined together, and a quality-aware correcting model was used for amplicon data to remove noise, chimeras and residual PhiX reads, dereplicate DNA reads and call ASVs (Callahan et al., 2017). Sequences clustered into 438 ASVs after filtering, which were then classified taxonomically against the SILVA database (v132) (Quast et al., 2013). Uncharacterized ASVs classified as not assigned (NA) at the phylum level (i.e. ASVs not assigned to any known species at the phylum level) or appearing in less than three samples were removed using a prevalence threshold<number of samples *0.05=18.60.

The unsupervised prevalence threshold value was selected at 5% of all samples. Alpha diversity metrics to measure the richness of the communities within samples were performed using the estimate richness function within the phyloseq package (McMurdie and Holmes, 2013). Tree maps were generated with the treemapify package (https://cran.r-project.org/web/packages/treemapify/index.html) to show the relative abundance between different groups and time points. PCoA using Bray-Curtis dissimilarity were implemented using proportional normalized data to reveal differences between various groups or time points. The ‘Adonis’ feature from vegan, measured as R^2^, was used to assess whether sample grouping by metadata factor accounted for inter-sample differences (https://CRAN.R-project.org/package=vegan). Three methods were tested: Unfirac (Lozupone and Knight, 2005), Bray-Curtis and Euclidean were implemented to calculate the distances among these samples. A significance *P*-value was generated by comparing that obtained by R^2^ to that obtained from 1000 random data permutations. F and B contributions were evaluated by pairwise comparison between samples from averaged SOD1^G93A^ versus WT mice using a Mann–Whitney test for each F and B species that contributed >1% to abundance. *P*-values identified statistically significant differences in F and B contributions (Table S3).

Differential abundance analysis was done using the DESeq2 package with *P*<0.05 (Love et al., 2014). LEfSe was performed to identify significant biomarkers with LDA>2 and *P*<0.05 (Segata et al., 2011). All ASVs were assigned to an archived and publicly available reference sequence (bacterial genome) in the SILVA database. Next, each matched bacterial genome from SILVA was transformed into a functional profile using Tax4Fun2 (Asshauer et al., 2015; Wemheuer et al., 2018 preprint). This program infers functional profiles from bacterial genomes using predictive tools applied to genome sequences. It identifies open reading frames and deduces protein expression by normalizing to the number of 16S rRNA genes within each genome. Although not as comprehensive and exhaustive as metagenomic shotgun sequencing, Tax4Fun2 provides a good approximation of functional profiles using 16S sequencing data that has already been collected to evaluate microbiome diversity (Asshauer et al., 2015; Wemheuer et al., 2018 preprint). Finally, the functional profiles were analyzed by multiple testing of KEGG pathway abundance using the most updated KEGG database (Kanehisa et al., 2012). The significant pathways were displayed in a heatmap (levels 1 and 2).

#### Data analysis for OTUs

The 16S rRNA gene sequence data was processed and analyzed using the software package mothur (Kozich et al., 2013; Schloss, 2009; Schloss et al., 2009). OTUs were obtained based on 97% sequence similarity. By calculating θ_YC_ distances, a metric that takes relative abundances of both shared and non-shared OTUs into account (Yue and Clayton, 2005), between communities and using AMOVA (Anderson, 2001), we tested whether there were statistically significant differences between the microbiota in SOD1^G93A^ versus control samples. OTUs were taxonomically classified by comparing sequences with a Ribosomal Database Project training set (Wang et al., 2007; Cole et al., 2014).

### Immunophenotyping analysis

#### Blood leukocyte collection

After opening the thoracic cavity, the volume of whole blood from the vena cava was measured using a 1 ml syringe (BD Biosciences) and then transferred to a BD Vacutainer^®^ blood collection tube (BD Biosciences) coated with 3.6 mg of EDTA. Red blood cells were lysed with 3 ml red blood cell lysis buffer [150 mM NH_4_Cl, 10 mM KHCO_3_, 0.1 mM EDTA (Thermo Fisher Scientific) with 13.8 mM HEPES (pH 7.2-7.5) (Thermo Fisher Scientific)] for 15 min on a rocker at room temperature. Leukocytes were pelleted [1000 rpm (300 ***g***), 10 min, 4°C, with brake], supernatant siphoned off, washed with flow cytometry buffer [1× PBS (Thermo Fisher Scientific), 2% fetal bovine serum (FBS) (Thermo Fisher Scientific), 0.1% NaN_3_], pelleted again (same conditions), and the wash cycle was repeated once more. In the final wash, cells were resuspended in 0.5 ml flow buffer, counted by hemocytometer (Hausser Scientific) and kept on ice until staining for flow cytometry.

#### Brain and spinal cord leukocyte collection

After whole blood collection from each mouse, their vascular system was flushed with sterile PBS to remove blood-borne leukocytes from peripheral tissue. The right brain hemisphere and the spinal cord were excised, each minced apart using surgical scissors and processed separately onwards. Tissue samples were incubated for 90 min at 37°C in supplemented RPMI [RPMI-1640 medium (Thermo Fisher Scientific)] with 5% FBS (Thermo Fisher Scientific), 50 and 100 µg ml^–1^ penicillin and streptomycin (Sigma-Aldrich), respectively, and 20 mg ml^–1^ *Clostridium histolyticum* collagenase (Sigma-Aldrich) with gentle mixing at 15 min intervals. The minced, digested tissue was placed in a 70 µm sterile nylon cell strainer (Corning) over a 50 ml conical tube (Corning) and dissociated further by grinding with a sterile 3 ml syringe plunger (BD Biosciences), and then washed twice with 10 ml supplemented RPMI. Cells were pelleted [1200 rpm (240 ***g***), 10 min, 4°C, with brake], supernatant siphoned off, and resuspended in 30% stock isotonic Percoll [90% Percoll (GE Healthcare) and 10% 10× HBSS without Ca^2+^ or Mg^2+^ (Thermo Fisher Scientific)]. The resuspended cells were gently layered onto 2 ml of 70% stock isotonic Percoll and spun at 500 ***g*** (30 min, 18°C, without brake). Leukocytes were transferred from the interphase to a clean 15 ml conical tube, washed with 10 ml RPMI, pelleted [1200 rpm (240 ***g***), 10 min, 4°C, with brake], supernatant siphoned off, and resuspended in 1 ml RPMI, counted by hemocytometer, and kept on ice until staining for flow cytometry.

#### Spleen leukocyte collection

Spleens were placed into a 70 µm sterile nylon cell strainer within the well of a 6-well plate (Corning) containing 3 ml supplemented RPMI. Each spleen was dissociated by grinding with a sterile 3 ml syringe plunger. The cell strainer was removed and the remaining cell suspension was transferred to a clean 15 ml conical tube. Cells were pelleted, supernatant aspirated, cells resuspended and then incubated for 10 min in 3 ml red blood cell lysis buffer. RPMI (10 ml) was added to each tube, and the leukocytes were pelleted, the media aspirated, and resuspended in 1 ml RPMI; cells were kept on ice until staining for flow cytometry.

#### Bone marrow collection

Bone marrow was flushed from the tibias with RPMI dispensed through a 26-gauge needle. The cells were drawn into a 10 ml syringe and dispersed via trituration through a 21-gauge needle; they were then resuspended in red blood cell lysis buffer, pelleted, washed, pelleted again and resuspended in 1 ml RPMI; cells were kept on ice until staining for flow cytometry.

#### Flow cytometry analysis

Cells were washed and resuspended at a density of ≤10^6^ cells/25 µl flow buffer, and Fc receptors were blocked by adding 10 µg ml^–1^ TruStain FcX™ blocking solution (BioLegend). Cells were then stained in a 50 µl final volume in 96-well round-bottom plates (Corning) covered for 30 min at 4°C and washed twice with flow buffer (1× PBS, 2% FBS, 1% sodium azide) before resuspension in 150 µl of BD™ Stabilizing Fixative (BD Biosciences) and transfer to polystyrene tubes (12×75 mm) (Becton Dickinson). A total of 0.5×10^5^ to 1×10^5^ events were acquired on a BD FACSCanto™ flow cytometer with FACSDiva™ software (BD Biosciences) and analyzed by FlowJo. Fluorophore-conjugated antibodies were APC-CD45 (BD Biosciences) and BV421-CD11c, BV421-CD8, FITC-Ly6C, PE-F4/80, PE-CD31, PerCP-CD3, PerCP-CD19, PE/Cy7-Ly6G, APC/Cy7-CD11b, and APC/Cy7-CD4 (BioLegend) (antibody details in Table S6).

### Global DNA 5mC and 5hmC analysis

The left-brain hemispheres and ileum were harvested from 37-day-old and ES SOD1^G93A^ and WT littermate mice and snap frozen. Genomic DNA was extracted using a DNeasy Blood and Tissue Kit (Qiagen), and global 5mC and 5hmC were determined in duplicate using a colorimetric enzyme-linked immunosorbent assay (ELISA) MethylFlash (methylation, P-1034-96; hydroxymethylation, P-1036-96) DNA Quantification Kits (EpiGentek). A Fluoroskan Ascent microplate reader recorded absorbance at 450 nm (Thermo Fisher Scientific) and the percentage of global 5mC and 5hmC was expressed as the mean±s.e.m. Data were analyzed using two-tailed Student's *t*-test.

### Correlation matrix

For correlation of ALS parameters, the data were analyzed by the University of Michigan Bioinformatics Core (https://brcf.medicine.umich.edu/cores/bioinformatics-core/). Only microbiome data from fecal pellets were used, as they had more time points. Data were first cleaned and null parameters, including undetectable microbiome OTUs, were removed from the dataset. The data were then organized into a single matrix with metadata as columns. Each row of the matrix consisted of all compiled data from a single mouse at a single time point. R (www.r-project.org) was then used to calculate the Pearson correlation coefficient between data columns over time. Cor.mtest from the corrplot package was then used to calculate *P*-values and generate the correlative heatmap. Temporal changes between two parameters that both increased (or decreased) were annotated as positive correlations (blue color in the heatmap), whereas temporal changes in parameters that saw inverse trends (i.e. one increased, one decreased) were annotated as negative correlations (red color in the heatmap). *P*-values were adjusted using Bonferroni correction to control the family-wise error rate. Bonferroni was used instead of Benjamini-Hochberg to increase the stringency of the analysis and confidence in the discovered correlations.

### Statistical analysis

All graphing and statistical calculations, except for microbiome data, were performed using Prism (version 7.01, GraphPad). Details for the analysis of microbiome data are included in the Microbiome Analysis subsection. For neuromuscular and immune phenotyping, all data were normally distributed and two-tailed Student's *t*-test with multiple comparisons were used to determine significance, with details provided in the figure legends. A *P*-value of 0.01 was taken to be statistically significant to account for multiple comparisons. Data are expressed as mean±s.e.m.

This article is part of a special collection ‘A Guide to Using Neuromuscular Disease Models for Basic and Preclinical Studies’, which was launched in a dedicated issue guest edited by Annemieke Aartsma-Rus, Maaike van Putten and James Dowling. See related articles in this collection at http://dmm.biologists.org/collection/neuromuscular.

## Supplementary Material

Supplementary information
