# Supplementary material for: Temporal evolution of the microbiome, immune system and epigenome with disease progression in ALS mice
Source: Dis Model Mech. 2019 Nov 15;13(2):dmm041947. doi: 10.1242/dmm.041947 (PMC6906635; doi:10.1242/dmm.041947)
Supplement: Supplementary information [file dmm-13-041947-s1.pdf]

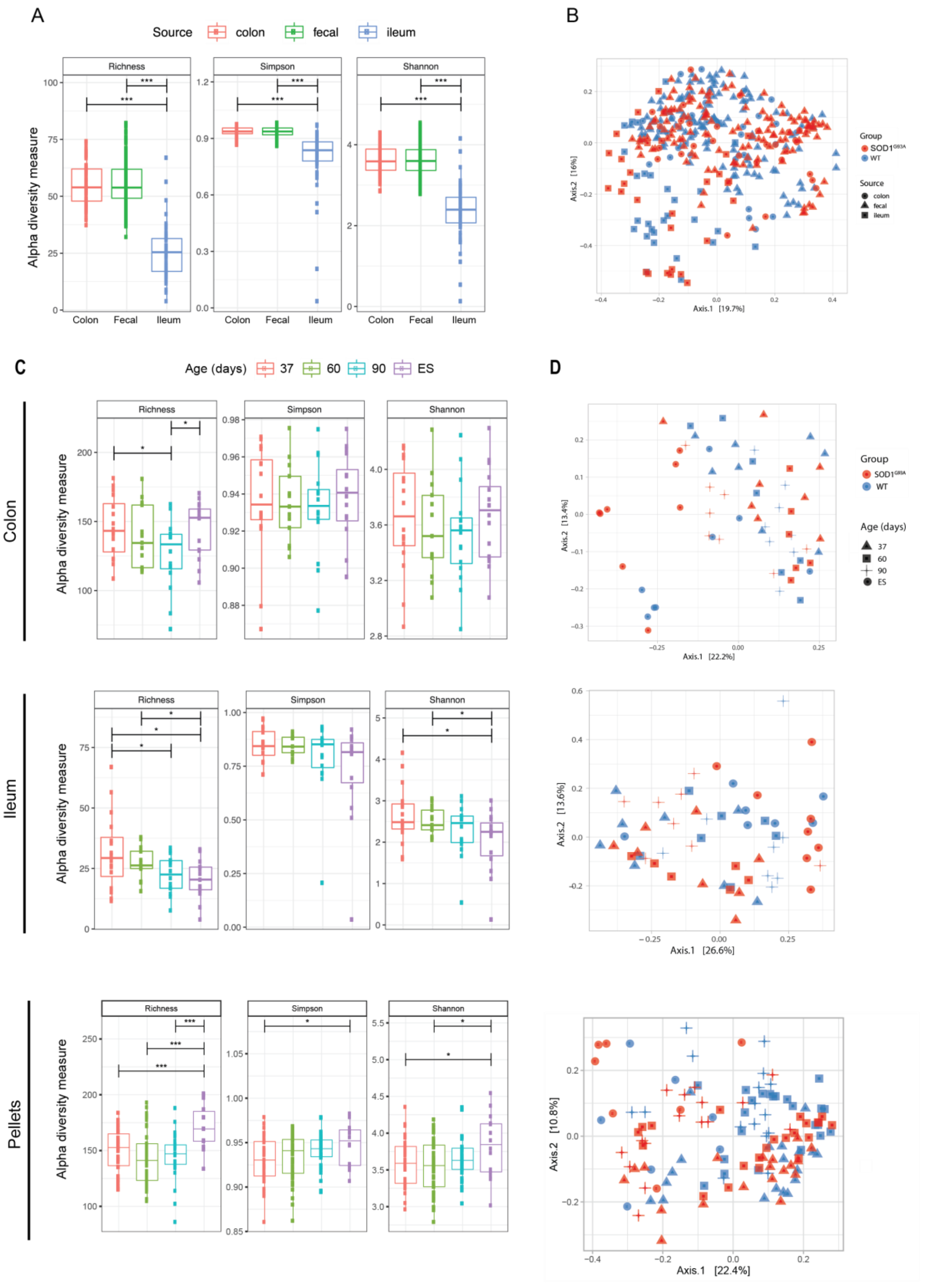

Fig. S1. Age, intestinal niche, and disease state determine overall bacterial diversity. (A) Overall alpha diversity by ASVs of pooled SOD1^{G93A} and WT samples was determined by richness, Simpson, and Shannon indices for colon (red), fecal pellets (green), and ileum (blue). Data are presented by box plots with horizontal line indicating the median for each data set. The x-axis represents the grouping, the y-axis represents the distance, and boxes

Disease Models & Mechanisms • Supplementary information

of different colors represent each group. ****P*<0.001, by ANOVA; n=16 mice per group for 37 and 90 days and ES; n=14 for 60 day-old mice. (B) PCoA plots representing beta diversity of bacterial communities from colon, fecal pellets, and ileum samples of $SOD1^{G93A}$ (red) and WT (blue) mice. (C) Overall alpha diversity by ASVs of pooled $SOD1^{G93A}$ and WT samples was determined by richness, Simpson, and Shannon indices in colon (top), ileum (middle), and fecal pellets (bottom) at 30 (red), 60 (green), 90 (blue) days, and ES (purple). Data are presented by box plots with horizontal line indicating the median for each data set. The x-axis represents the grouping, the y-axis represents the distance, and boxes of different colors represent each group. **P*<0.01, by ANOVA; n=16 mice per group for 37 and 90 days and ES; n=14 for 60 day-old mice. (D) PCoA plots representing beta diversity of bacterial communities from colon, fecal pellets, and ileum samples of $SOD1^{G93A}$ (red) and WT (blue) mice over time [30 (triangle), 60 (square), 90 (cross) days, and ES (circle)].

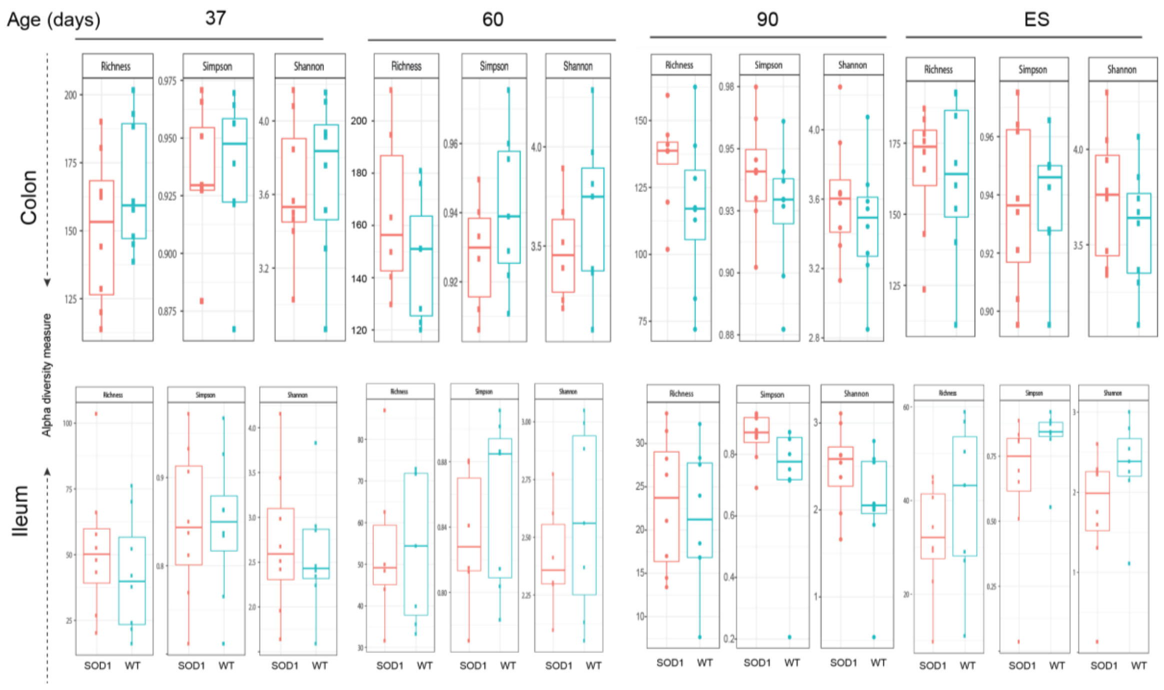

Fig. S2. Alpha diversity is not altered in the colon and ileum of SOD1^{G93A} mice. Alpha diversity for colon and ileum was determined by richness, Simpson, and Shannon indices in SOD1^{G93A} (red) and WT (blue) mice. Data are presented by box plots with horizontal line indicating the median for each data set. The number of animals (n) per group (SOD1^{G93A}/WT) at each time point was: colon, 37 days=8/8, 60 days=7/6, 90 days=8/8, ES=8/8; ileum, 37 days=8/8, 60 days=7/6, 90 days=8/8, ES=7/8.

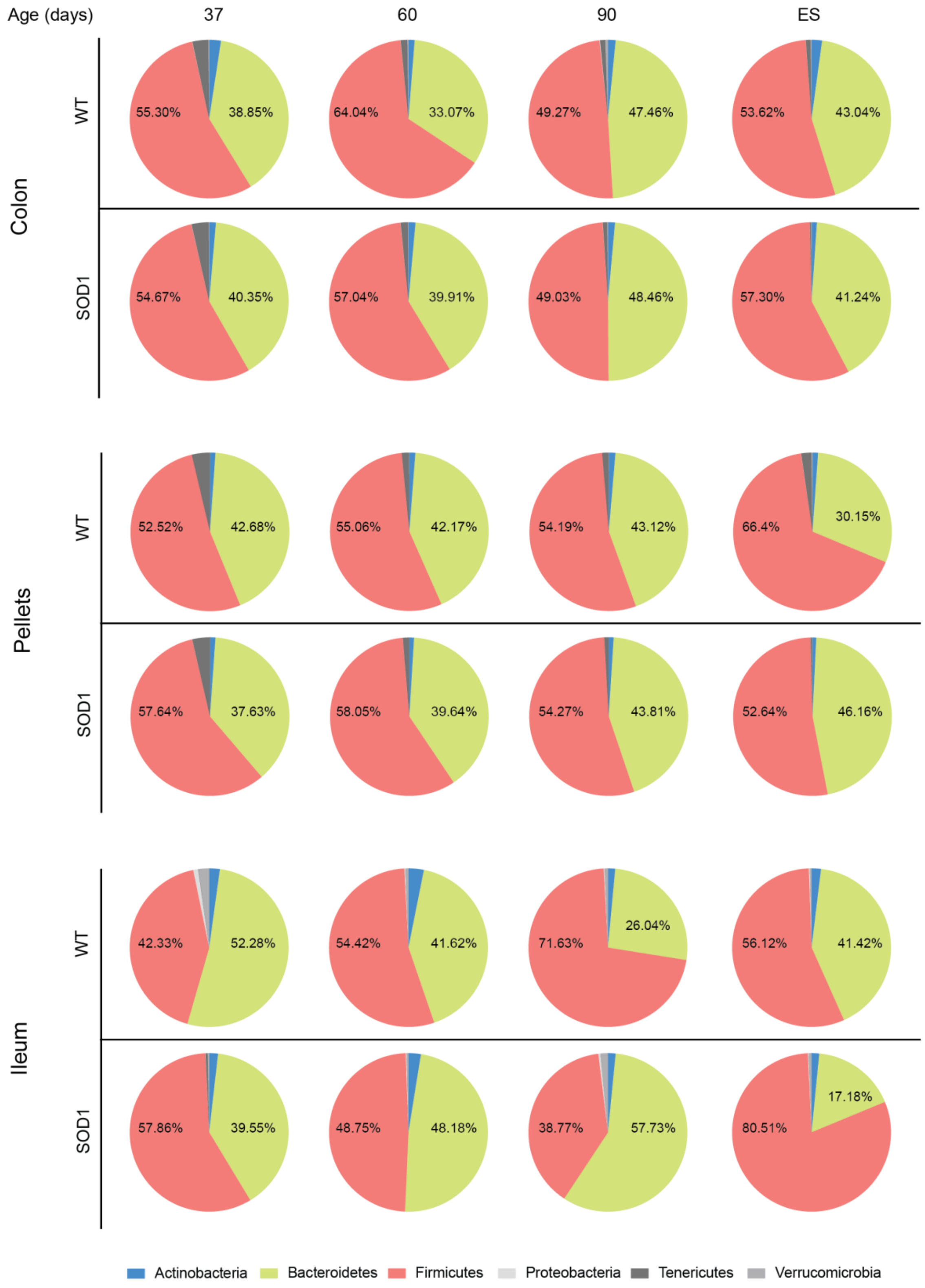

Fig. S3. Firmicutes (F) to Bacteroidetes (B) proportions in SOD1^{G93A} versus WT mice. F (red) and B (green) proportions represented as pie charts of cumulative relative abundance for all samples at each time point 37, 60, and 90 days and ES in colon (top), pellets (middle), and ileum (bottom). Pairwise comparison between samples from SOD1^{G93A} versus WT mice was performed using a Mann-Whitney test for each F and B species that contributed >1% to abundance to evaluate *P*-values (Table S3).

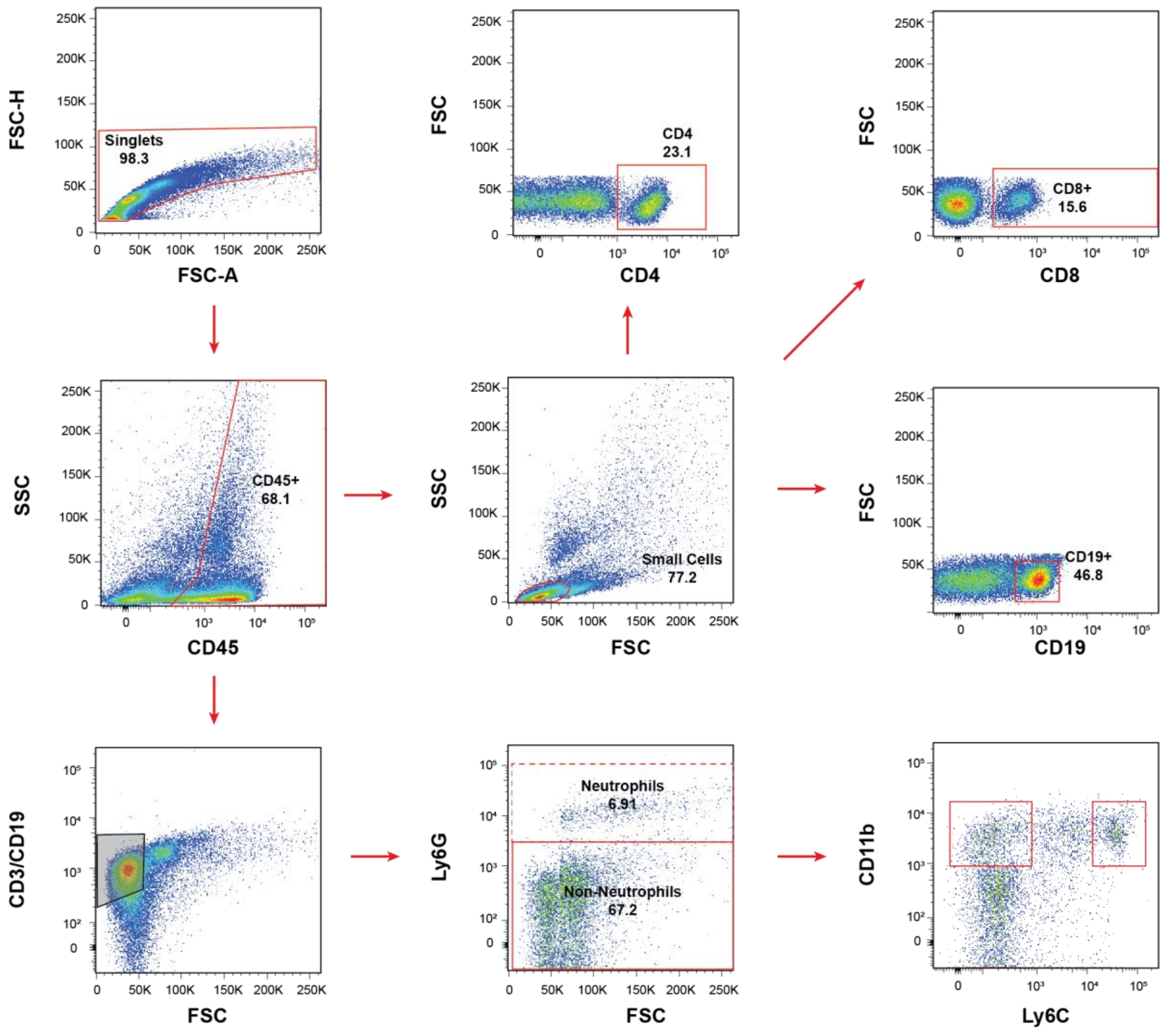

Fig. S4. Gating strategy for immune cells from blood. Staining was performed using CD45, CD3, CD4, CD8, CD19, Ly6C, Ly6G, and CD11b. Within CD45^{+} cells, CD4 T cells (CD3^{+} CD4^{+} CD8^{-}), CD8 T cells (CD3^{+} CD4^{-} CD8^{+}), or B cells (CD3^{-} CD19^{+}) were identified. Myeloid cells were identified using side scatter (SSC), CD11b, Ly6C, Ly6G, and CD45 for neutrophils (SSC^{high} CD11b^{+} Ly6G^{+}), and monocytes (CD45^{high} CD11b^{+} Ly6G^{-}, Ly6C$^{\pm}$).

Disease Models & Mechanisms • Supplementary information

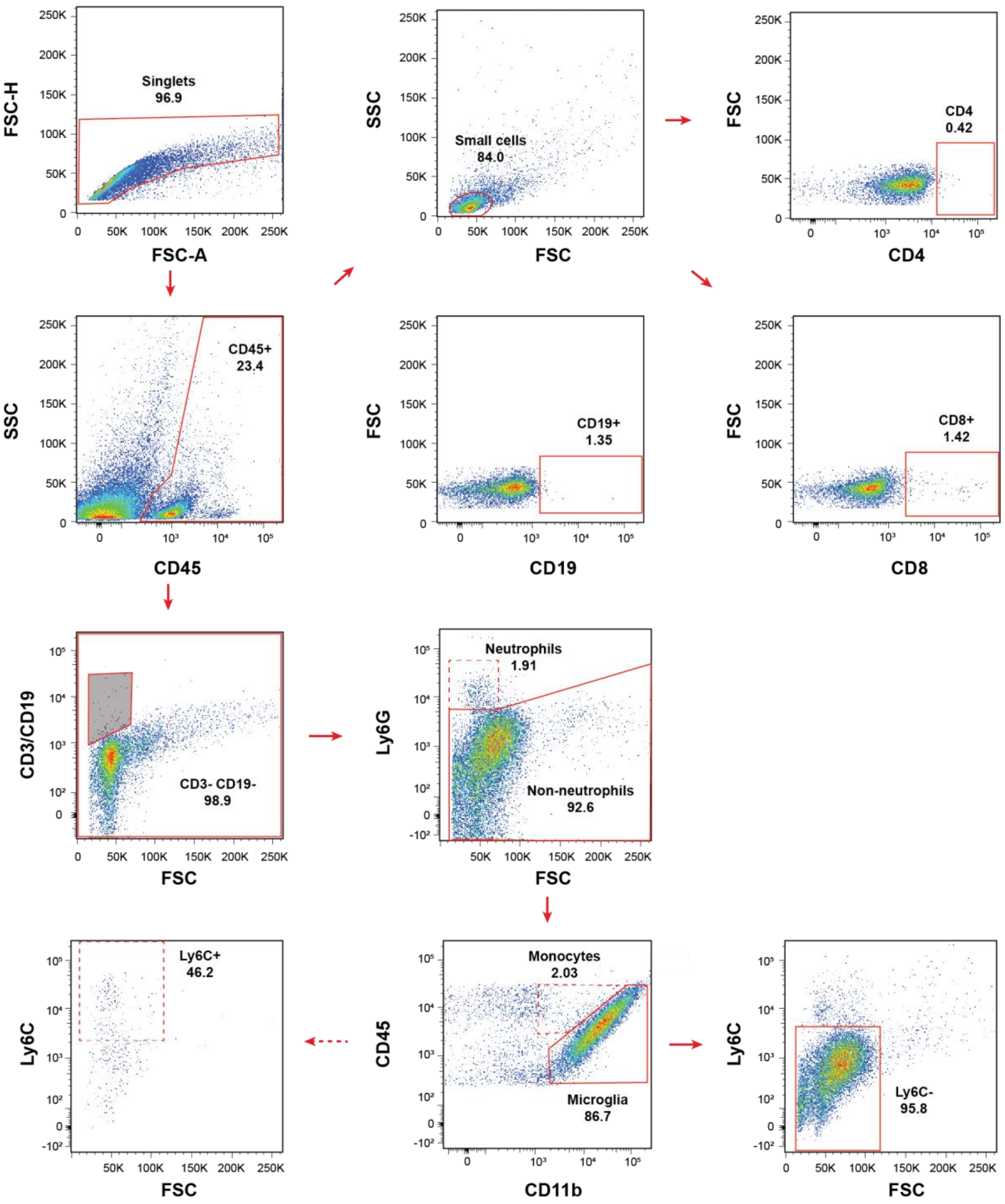

Fig. S5. Gating strategy for immune cells from brain and spinal cord tissue. Staining was performed using CD45, CD3, CD4, CD8, CD19, Ly6C, Ly6G, and CD11b. Within CD45$^+$ cells, CD4 T cells (CD3$^+$ CD4$^+$ CD8$^-$), CD8 T cells (CD3$^+$ CD4$^-$ CD8$^+$), or B cells (CD3$^-$ CD19$^+$) were identified. Myeloid cells were identified using side scatter (SSC), CD11b, Ly6C, Ly6G, and CD45 for neutrophils (SSC^{high} CD11b$^+$ Ly6G$^+$), Ly6C$^+$ monocytes (CD45^{high} CD11b$^+$ Ly6G$^-$, Ly6C$^+$), and microglia (CD45^{mid} CD11b$^+$ Ly6G$^-$).

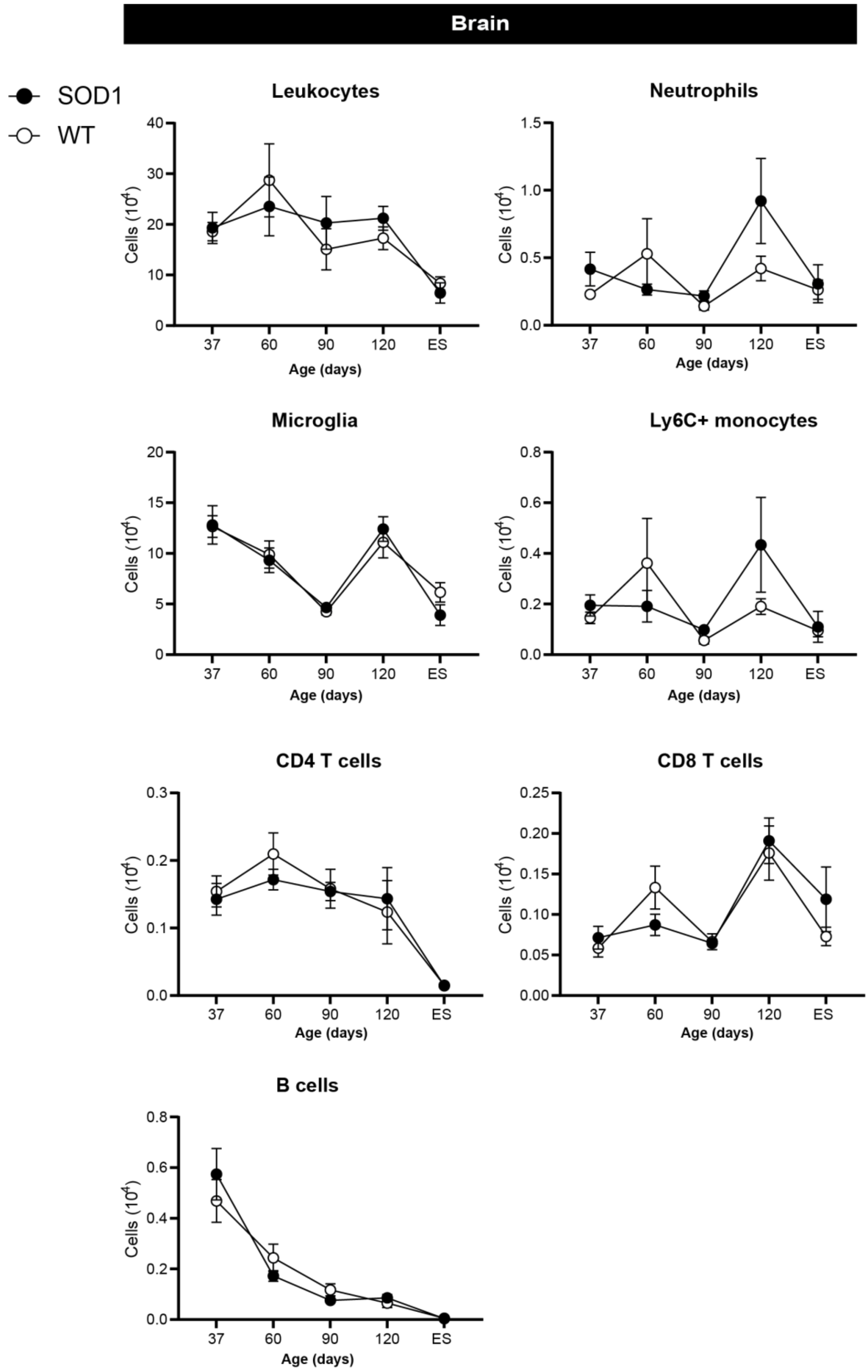

Fig. S6. Immune cell populations in the brain are not significantly altered during ALS. Immune cell populations were assessed in $SOD1^{G93A}$ (black) and WT (white) mice aged 37, 60, 90, and 120 days and at ES in brain tissue. Statistical analysis by two-tailed Student's t-test with multiple comparisons; the number of animals (n) per group ($SOD1^{G93A}$/WT) at each time point was: 37 days=8/8, 60 days=7/8, 90 days=8/8, 120 days=8/8, ES=8/7; data expressed as mean ± s.e.m.

Disease Models & Mechanisms • Supplementary information

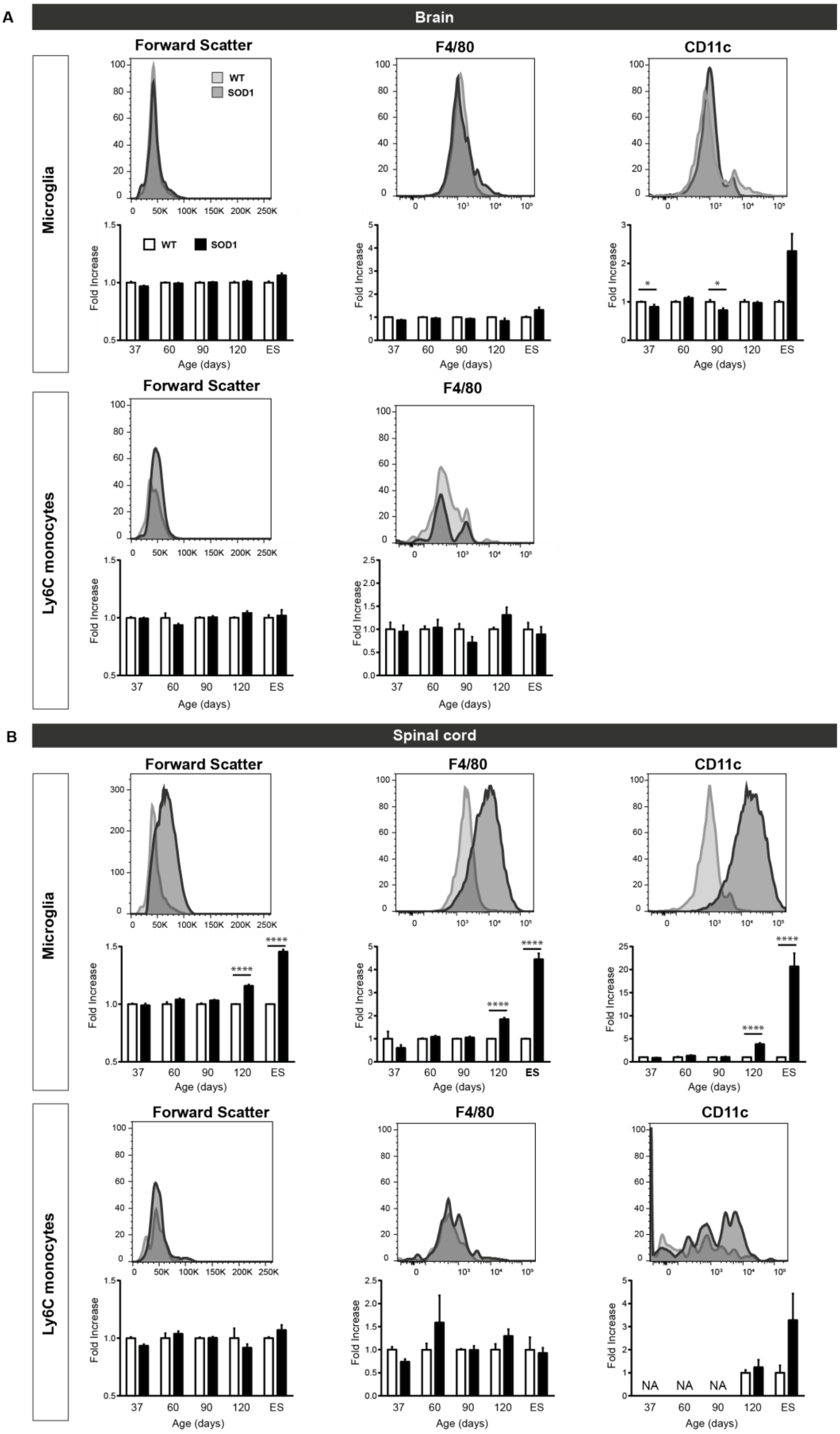

Disease Models & Mechanisms • Supplementary information

Fig. S7. Myeloid cells in the spinal cord are more activated during late-stage ALS. Flow cytometer histograms of forward scatter (size), F4/80, and CD11c expression levels were used to assess microglia and Ly6C^{+} monocyte activation in (A) brain and (B) spinal cord tissue in SOD1^{G93A} (dark grey) and WT (light grey) mice. Displayed histograms are representative plots from ES mice. Histograms were used to generate fold-increase in forward scatter and F4/80, and CD11c expression in SOD1^{G93A} (black) and WT (white) mice aged 37, 60, 90, and 120 days and at ES in (A) brain and (B) spinal cord tissue. **P*<0.01, *****P*<0.0001, by two-tailed Student's t-test with multiple comparisons; the number of animals (n) per group (SOD1^{G93A}/WT) at each time point was: 37 days=8/8, 60 days=7/8, 90 days=8/8, 120 days=8/8, ES=8/7; data expressed as mean ± s.e.m.

.

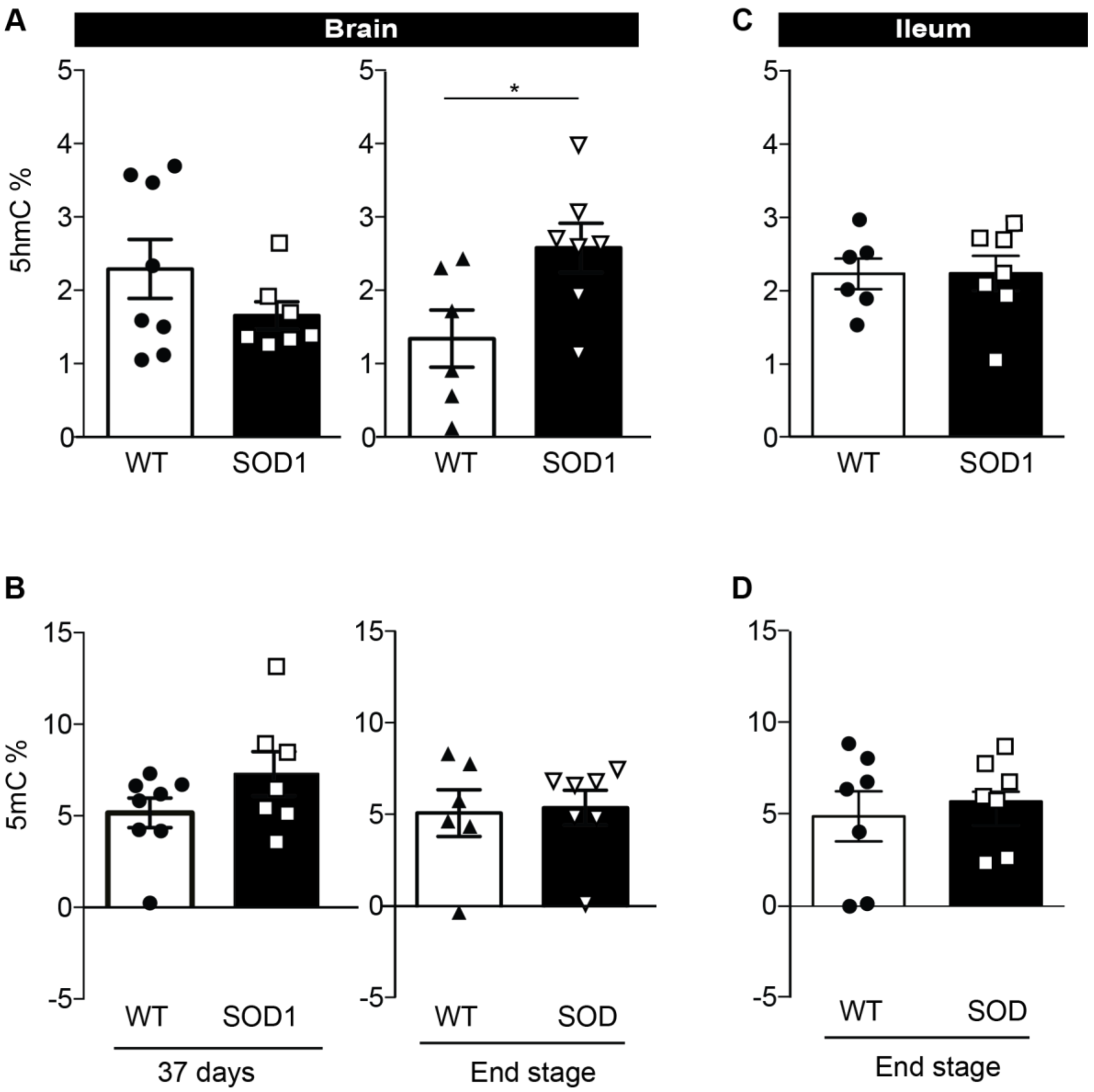

Fig. S8. Global hydroxymethylation (5hmC) but not methylation (5mC) is altered in the CNS during ALS progression. Genomic DNA from 37 day-old or ES brain (A, B) or ileum (C, D) from SOD1^{G93A} (white) or WT (black) mice was assessed for global 5hmC (A, C) or 5mC (B, D) by a colorimetric ELISA assay; **P*<0.05, by Student's t-test; the number of animals (n) per group (SOD1^{G93A}/WT) at each time point for 5hmC was: ileum at ES=7/6; brain at 37 days=7/8, ES=7/6. The number of animals (n) per group (SOD1^{G93A}/WT) at each time point for 5mC was: ileum at ES=7/7; brain at 37 days=7/8, ES=7/6; data expressed as mean ± s.e.m. of percent (% of total DNA).

Disease Models & Mechanisms • Supplementary information

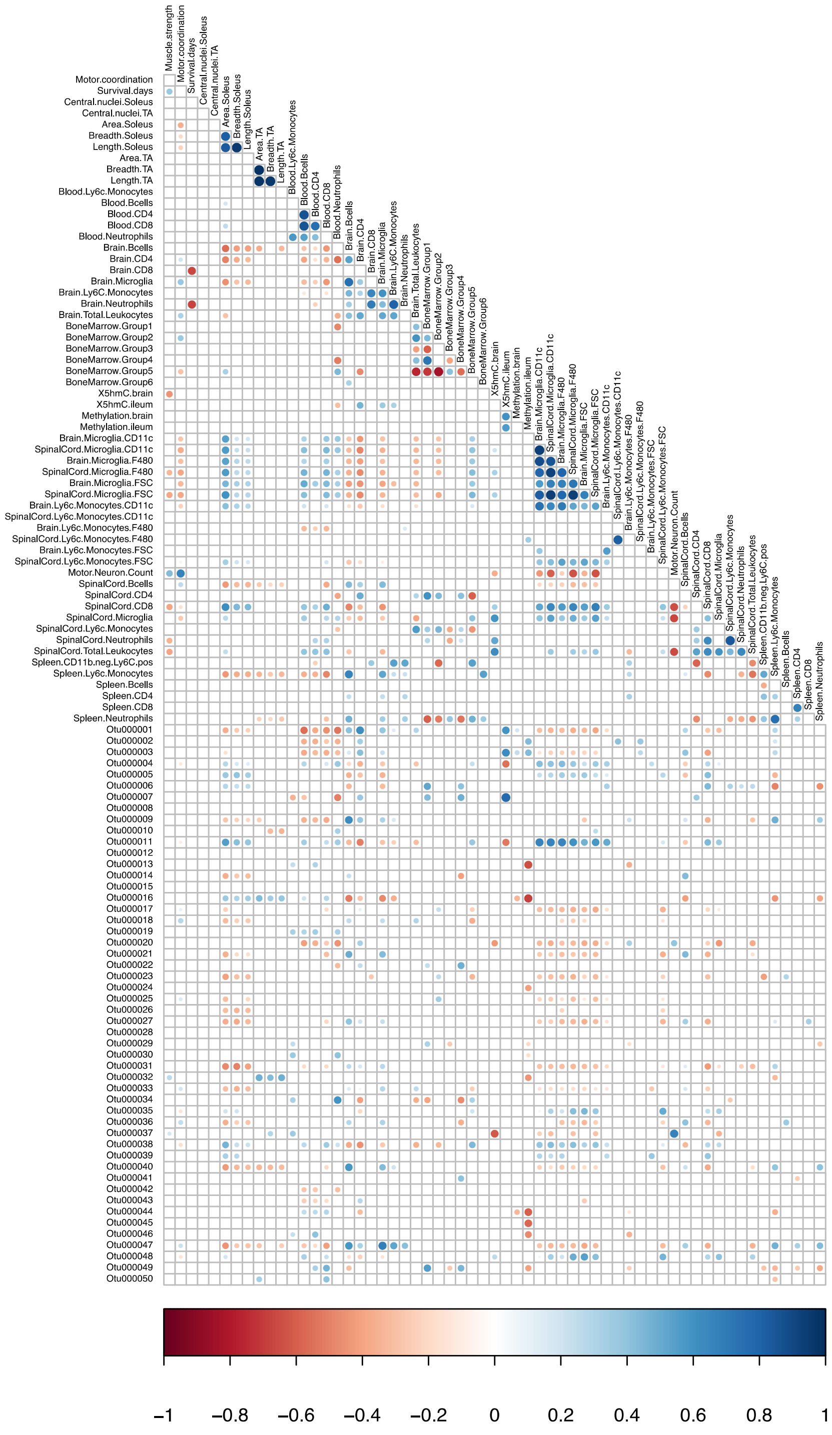

Fig. S9. Specific microbiome, epigenome, and immune changes are correlated in ALS. The correlation for the top 50 OTUs with immunophenotype, neuromuscular status, and DNA methylation is shown. The analysis was performed using Pearson correlation. Significant correlations are plotted as a circle at the intersection between correlated parameters along the y-axis with parameters on the diagonal. Circles are color coded on a scale of highly positively correlated (1, blue) and highly negatively correlated (-1, red).

Table S1. Quantification of beta diversity of bacterial communities in ileum, colon, and fecal pellets in SOD1^{G93A} versus WT mice using ASVs by phylogenetic (UniFrac, UF) and non-phylogenetic (Euclidean, E; Bray-Curtis, BC) methods. Asterisks denote *P*-values less than 0.05.

Age (days)	Method	Ileum *P*-values	Colon *P*-values	Fecal pellets *P*-values
37	E	0.492	0.905	0.307
	UF	0.411	0.579	0.139
	BC	0.743	0.823	0.245
45	E			0.246
	UF			0.030*
	BC			0.050
60	E	0.141	0.119	0.011*
	UF	0.669	0.034*	0.042*
	BC	0.163	0.097	0.001*
75	E			0.109
	UF			0.018*
	BC			0.050
90	E	0.020*	0.004*	0.010*
	UF	0.380	0.034*	0.020*
	BC	0.013*	0.011*	0.010*
105	E			0.132
	UF			0.092
	BC			0.079
120	E			0.090
	UF			0.016*
	BC			0.056
135	E			0.301
	UF			0.010*
	BC			0.130
ES	E	0.294	0.089	0.245
	UF	0.106	0.044*	0.077
	BC	0.197	0.036*	0.106

ASVs, amplicon sequence variants.

Table S2. Quantification of beta diversity of bacterial communities in ileum, colon, and fecal pellets in SOD1[G93A] versus WT mice using OTUs and AMOVA. Asterisks denote *P*-values less than 0.05.

Age (days)	Ileum *P*-values	Colon *P*-values	Fecal pellets *P*-values
37	0.679	0.813	0.515
45			0.185
60	0.058	0.069	0.014*
75			0.040*
90	0.016*	0.001*	0.006*
105			0.191
120			0.118
135			0.188
ES	0.100	0.128	0.217

AMOVA, analysis of molecular variance; ES, end-stage; OTUs, operational taxonomic units.

Table S3. F and B contributions across ileum, colon, and fecal pellets in SOD1^{G93A} versus WT mice. *P*-values were evaluated to identify statically significant differences by Mann-Whitney test for each F and B species that contributed >1% to abundance. Asterisks denote P-values less than 0.05.

	Age (days)	Firmicutes *P*-values	Bacteroidetes *P*-values
Ileum	37	0.69	0.76
	60	0.47	0.33
	90	0.57	0.18
	ES	0.05	0.03*
Colon	37	0.64	0.74
	60	0.27	0.02*
	90	0.76	0.36
	ES	0.19	0.60
Pellets	37	0.02*	0.77
	60	0.04*	0.80
	90	0.10	0.53
	ES	0.67	0.46

Disease Models & Mechanisms • Supplementary information

Table S4. Fifty most populated OTUs used for the correlation analysis. The number of species within each OTU (size) and taxonomy listed in order of kingdom, phylum, class, order, family, genus, species and the number of species.

OTU	Size	Taxonomy
OTU000001	883802	Bacteria(100);Bacteroidetes(100);Bacteroidia(100);Bacteroidales(100);Porphyromonadaceae(100);unclassified(100)
OTU000002	590546	Bacteria(100);Firmicutes(100);Bacilli(100);Lactobacillales(100);Lactobacillaceae(100);Lactobacillus(100)
OTU000003	444608	Bacteria(100);Bacteroidetes(100);Bacteroidia(100);Bacteroidales(100);Porphyromonadaceae(100);unclassified(100)
OTU000004	277552	Bacteria(100);Bacteroidetes(100);Bacteroidia(100);Bacteroidales(100);Porphyromonadaceae(100);unclassified(100)
OTU000005	277267	Bacteria(100);Firmicutes(100);Erysipelotrichia(100);Erysipelotrichales(100);Erysipelotrichaceae(100);Turicibacter(100)
OTU000006	262866	Bacteria(100);Bacteroidetes(100);Bacteroidia(100);Bacteroidales(100);Porphyromonadaceae(100);unclassified(100)
OTU000007	261009	Bacteria(100);Bacteroidetes(100);Bacteroidia(100);Bacteroidales(100);Porphyromonadaceae(100);unclassified(100)
OTU000008	207013	Bacteria(100);Firmicutes(100);Clostridia(100);Clostridiales(100);Lachnospiraceae(100);unclassified(100)
OTU000009	176647	Bacteria(100);Firmicutes(100);Bacilli(100);Lactobacillales(100);Lactobacillaceae(100);Lactobacillus(100)
OTU000010	147779	Bacteria(100);Bacteroidetes(100);Bacteroidia(100);Bacteroidales(100);Porphyromonadaceae(100);unclassified(100)
OTU000011	116171	Bacteria(100);Bacteroidetes(100);Bacteroidia(100);Bacteroidales(100);Porphyromonadaceae(100);Barnesiella(100)
OTU000012	99899	Bacteria(100);Firmicutes(100);Clostridia(100);Clostridiales(100);Lachnospiraceae(100);unclassified(100)
OTU000013	96228	Bacteria(100);Firmicutes(100);Clostridia(100);Clostridiales(100);Ruminococcaceae(100);Oscillibacter(100)
OTU000014	84891	Bacteria(100);Firmicutes(100);Clostridia(100);Clostridiales(100);Lachnospiraceae(100);unclassified(100)
OTU000015	84695	Bacteria(100);Firmicutes(100);Clostridia(100);Clostridiales(100);Clostridiaceae_1(100);Clostridium_sensu_stricto(100)
OTU000016	73849	Bacteria(100);Firmicutes(100);Clostridia(100);Clostridiales(100);Lachnospiraceae(100);unclassified(100)

OTU000017	67926	Bacteria(100);unclassified(100);unclassified(100);unclassified(100);unclassified(100);unclassified(100)
OTU000018	67533	Bacteria(100);Firmicutes(100);Clostridia(100);Clostridiales(100);Lachnospiraceae(100);unclassified(98)
OTU000019	63873	Bacteria(100);Firmicutes(100);Clostridia(100);Clostridiales(100);Lachnospiraceae(100);unclassified(100)
OTU000020	59084	Bacteria(100);Bacteroidetes(100);Bacteroidia(100);Bacteroidales(100);Porphyromonadaceae(100);unclassified(100)
OTU000021	58349	Bacteria(100);Firmicutes(100);Clostridia(100);Clostridiales(100);Lachnospiraceae(100);unclassified(100)
OTU000022	56845	Bacteria(100);Bacteroidetes(100);Bacteroidia(100);Bacteroidales(100);Porphyromonadaceae(100);unclassified(100)
OTU000023	55850	Bacteria(100);Firmicutes(100);Clostridia(100);Clostridiales(100);Lachnospiraceae(100);unclassified(100)
OTU000024	54799	Bacteria(100);Firmicutes(100);Clostridia(100);Clostridiales(100);Lachnospiraceae(100);Clostridium_XlVa(85)
OTU000025	53981	Bacteria(100);Firmicutes(100);Clostridia(100);Clostridiales(100);Lachnospiraceae(100);unclassified(100)
OTU000026	52217	Bacteria(100);Firmicutes(100);Clostridia(100);Clostridiales(100);unclassified(100);unclassified(100)
OTU000027	51049	Bacteria(100);Firmicutes(100);Clostridia(100);Clostridiales(100);Lachnospiraceae(100);unclassified(100)
OTU000028	50139	Bacteria(100);Bacteroidetes(100);Bacteroidia(100);Bacteroidales(100);Porphyromonadaceae(100);unclassified(100)
OTU000029	48349	Bacteria(100);Firmicutes(100);Clostridia(100);Clostridiales(100);Ruminococcaceae(100);unclassified(98)
OTU000030	44587	Bacteria(100);Firmicutes(100);Clostridia(100);Clostridiales(100);Ruminococcaceae(100);unclassified(70)
OTU000031	43622	Bacteria(100);Firmicutes(100);Clostridia(100);Clostridiales(100);Lachnospiraceae(100);unclassified(100)
OTU000032	42111	Bacteria(100);Firmicutes(100);Clostridia(100);Clostridiales(100);Lachnospiraceae(100);unclassified(100)
OTU000033	41423	Bacteria(100);Firmicutes(100);Clostridia(100);Clostridiales(100);Lachnospiraceae(100);unclassified(100)
OTU000034	40786	Bacteria(100);Firmicutes(100);Clostridia(100);Clostridiales(100);Lachnospiraceae(100);unclassified(100)
OTU000035	39264	Bacteria(100);Firmicutes(100);Erysipelotrichia(100);Erysipelotrichales(100);Erysipelotrichaceae(100);unclassified(100)

Disease Models & Mechanisms • Supplementary information

OTU000036	38613	Bacteria(100);Tenericutes(100);Mollicutes(100);Anaeroplasmatales(100);Anaeroplasmataceae(100);Anaeroplasma(100)
OTU000037	35714	Bacteria(100);Firmicutes(100);Clostridia(100);Clostridiales(100);Ruminococcaceae(100);unclassified(100)
OTU000038	35176	Bacteria(100);Firmicutes(100);Clostridia(100);Clostridiales(100);Lachnospiraceae(100);unclassified(100)
OTU000039	34422	Bacteria(100);Bacteroidetes(100);Bacteroidia(100);Bacteroidales(100);Porphyromonadaceae(100);unclassified(100)
OTU000040	31829	Bacteria(100);Firmicutes(100);Clostridia(100);Clostridiales(100);Lachnospiraceae(100);unclassified(100)
OTU000041	30159	Bacteria(100);Firmicutes(100);Clostridia(100);Clostridiales(100);Lachnospiraceae(100);unclassified(100)
OTU000042	29472	Bacteria(100);Firmicutes(100);Clostridia(100);Clostridiales(100);Peptostreptococcaceae(100);Clostridium_XI(98)
OTU000043	29186	Bacteria(100);Firmicutes(100);Clostridia(100);Clostridiales(100);Lachnospiraceae(100);unclassified(100)
OTU000044	28000	Bacteria(100);Firmicutes(100);Clostridia(100);Clostridiales(100);Ruminococcaceae(100);Oscillibacter(100)
OTU000045	27526	Bacteria(100);Firmicutes(100);Clostridia(100);Clostridiales(100);Lachnospiraceae(99);unclassified(99)
OTU000046	27445	Bacteria(100);Firmicutes(100);Clostridia(100);Clostridiales(100);Lachnospiraceae(100);unclassified(100)
OTU000047	27252	Bacteria(100);Actinobacteria(100);Actinobacteria(100);Coriobacteriales(100);Coriobacteriaceae(100);Asaccharobacter(88)
OTU000048	27049	Bacteria(100);Bacteroidetes(100);Bacteroidia(100);Bacteroidales(100);Porphyromonadaceae(100);unclassified(100)
OTU000049	24695	Bacteria(100);Firmicutes(100);Clostridia(100);Clostridiales(100);Lachnospiraceae(100);Johnsonella(100)
OTU000050	24126	Bacteria(100);Firmicutes(100);Clostridia(100);Clostridiales(100);Lachnospiraceae(100);unclassified(100)

OTUs, operational taxonomic units.

Disease Models & Mechanisms • Supplementary information

Table S5. Correlation analysis results of pathological processes in SOD1^{G93A} ALS mice with OTUs.

OTU	Correlation
OTU000037	Correlated positively with motor neuron density
OTU000032	Correlated positively with TA muscle size (area, breadth, length)
OTU000001	Correlated negatively with activated brain and spinal cord microglia, and with spinal cord CD8 T cell levels
OTU000020	
OTU000021	
OTU000023	
OTU000025	
OTU000027	
OTU000031	
OTU000040	
OTU000047	
OTU000004	Correlated positively with activated brain and spinal cord microglia, and with spinal cord CD8 T cell levels
OTU000005	
OTU000011	
OTU000035	
OTU000038	
OTU000048	
OTU000001	Correlated positively with global ileum 5hmC
OTU000003	
OTU000007	
OTU000004	Correlated negatively with global ileum 5hmC
OTU000011	
OTU000037	Correlated negatively with global brain 5hmC
OTU000002	Correlated positively with global ileum 5mC
OTU000003	
OTU000013	Correlated negatively with global ileum 5mC
OTU000016	
OTU000044	
OTU000045	

OTUs, operational taxonomic units; TA, tibialis anterior muscle.

Disease Models & Mechanisms • Supplementary information

Table S6. Antibody characteristics from MATERIALS AND METHODS section.

Antibody	Manufacturer	Catalog #	Batch	Reactivity	Clone	Isotype	Dilution	RRID	references
APC-CD45	BD Biosciences	559864	3354869	Mouse	30-F11	Rat	1:200	AB_398672	(Peng et al., 2014)
BV421-CD11c	BioLegend	117330	B180890	Mouse	N418	Armenian Hamster	1:100	AB_11219593	(Benson et al., 2007)
BV421-CD8	BioLegend	100738	B190821	Mouse	53-6.7	Rat	1:100	AB_11204079	(Ko et al., 2005)
FITC-Ly6C	BioLegend	128006	B193352	Mouse	HK1.4	Rat	1:100	AB_1186135	(Watson et al., 2015)
PE-F4/80	BioLegend	123110	B181891	Mouse	BM8	Rat	1:200	AB_893486	(Poeckel et al., 2009)
PE-CD31	BioLegend	102408	B163731	Mouse	390	Rat	1:200	AB_312903	(Greineder et al., 2013)
PerCP-CD3	BioLegend	100326	B187521	Mouse	145-2C11	Armenian Hamster	1:200	AB_893317	(Leo et al., 1987)
PerCP-CD19	BioLegend	115532	B173071	Mouse	6D5	Rat	1:200	AB_2072926	(Bankoti et al., 2010)
PE/Cy7-Ly6G	BioLegend	127618	B222650	Mouse	1A8	Rat	1:500	AB_1877261	(Tadagavadi and Reeves, 2010)
APC/Cy7-CD11b	BioLegend	101226	B191242	Mouse	M1/70	Rat	1:500	AB_830642	(Tan et al., 2006)
APC/Cy7-CD4	BioLegend	100414	B186911	Mouse	GK1.5	Rat	1:200	AB_312699	(Felix et al., 2007)
Dystrophin	Abcam	ab15277		Mouse, Rat, Dog, Human	Polyclonal	Rabbit	1:300	AB_301813	(Jelinkova et al., 2019)
Secondary, Alexa Fluor 488	Thermo Fisher Scientific	A-11034		Rabbit	Polyclonal	Goat	1:1000	AB_2576217	(Brendel et al., 2017)

APC, allophycocyanin; APC/Cy7, APC and Cy7 conjugate; BV421, Brilliant Violet 421™; Cy7, cyanine 7; FITC, fluorescein isothiocyanate; PE, R-phycoerythrin; PE/Cy7, PE and Cy7 conjugate; PerCP, peridinin-chlorophyll-protein.

BANKOTI, J., BURNETT, A., NAVARRO, S., MILLER, A. K., RASE, B. & SHEPHERD, D. M. 2010. Effects of TCDD on the fate of naive dendritic cells. *Toxicol Sci,* 115**,** 422-34.

BENSON, M. J., PINO-LAGOS, K., ROSEMBLATT, M. & NOELLE, R. J. 2007. All-trans retinoic acid mediates enhanced T reg cell growth, differentiation, and gut homing in the face of high levels of co-stimulation. *J Exp Med,* 204**,** 1765-74.

BRENDEL, M., KLEINBERGER, G., PROBST, F., JAWORSKA, A., OVERHOFF, F., BLUME, T., ALBERT, N. L., CARLSEN, J., LINDNER, S., GILDEHAUS, F. J., OZMEN, L., SUAREZ-CALVET, M., BARTENSTEIN, P., BAUMANN, K., EWERS, M., HERMS, J., HAASS, C. & ROMINGER, A. 2017. Increase of TREM2 during Aging of an Alzheimer's Disease Mouse Model Is Paralleled by Microglial Activation and Amyloidosis. *Front Aging Neurosci,* 9**,** 8.

FELIX, N. J., DONERMEYER, D. L., HORVATH, S., WALTERS, J. J., GROSS, M. L., SURI, A. & ALLEN, P. M. 2007. Alloreactive T cells respond specifically to multiple distinct peptide-MHC complexes. *Nat Immunol,* 8**,** 388-97.

GREINEDER, C. F., CHACKO, A. M., ZAYTSEV, S., ZERN, B. J., CARNEMOLLA, R., HOOD, E. D., HAN, J., DING, B. S., ESMON, C. T. & MUZYKANTOV, V. R. 2013. Vascular immunotargeting to endothelial determinant ICAM-1 enables optimal partnering of recombinant scFv-thrombomodulin fusion with endogenous cofactor. *PLoS One,* 8**,** e80110.

JELINKOVA, S., FOJTIK, P., KOHUTOVA, A., VILOTIC, A., MARKOVA, L., PESL, M., JURAKOVA, T., KRUTA, M., VRBSKY, J., GAILLYOVA, R., VALASKOVA, I., FRAK, I., LACAMPAGNE, A., FORTE, G., DVORAK, P., MELI, A. C. & ROTREKL, V. 2019. Dystrophin Deficiency Leads to Genomic Instability in Human Pluripotent Stem Cells via NO Synthase-Induced Oxidative Stress. *Cells,* 8.

KO, S. Y., KO, H. J., CHANG, W. S., PARK, S. H., KWEON, M. N. & KANG, C. Y. 2005. alpha-Galactosylceramide can act as a nasal vaccine adjuvant inducing protective immune responses against viral infection and tumor. *J Immunol,* 175**,** 3309-17.

LEO, O., FOO, M., SACHS, D. H., SAMELSON, L. E. & BLUESTONE, J. A. 1987. Identification of a monoclonal antibody specific for a murine T3 polypeptide. *Proc Natl Acad Sci U S A,* 84**,** 1374-8.

PENG, B., XIAO, J., WANG, K., SO, K. F., TIPOE, G. L. & LIN, B. 2014. Suppression of microglial activation is neuroprotective in a mouse model of human retinitis pigmentosa. *J Neurosci,* 34**,** 8139-50.

POECKEL, D., ZEMSKI BERRY, K. A., MURPHY, R. C. & FUNK, C. D. 2009. Dual 12/15- and 5-lipoxygenase deficiency in macrophages alters arachidonic acid metabolism and attenuates peritonitis and atherosclerosis in ApoE knock-out mice. *J Biol Chem,* 284**,** 21077-89.

TADAGAVADI, R. K. & REEVES, W. B. 2010. Endogenous IL-10 attenuates cisplatin nephrotoxicity: role of dendritic cells. *J Immunol,* 185**,** 4904-11.

TAN, S. L., ZHAO, J., BI, C., CHEN, X. C., HEPBURN, D. L., WANG, J., SEDGWICK, J. D., CHINTALACHARUVU, S. R. & NA, S. 2006. Resistance to experimental autoimmune encephalomyelitis and impaired IL-17 production in protein kinase C theta-deficient mice. *J Immunol,* 176**,** 2872-9.

WATSON, N. B., SCHNEIDER, K. M. & MASSA, P. T. 2015. SHP-1-dependent macrophage differentiation exacerbates virus-induced myositis. *J Immunol,* 194**,** 2796-809.
